# Fuzheng Jiedu formula attenuates acute pneumonia by coordinated regulation of macrophage NLRP3 inflammasome and neutrophil NETs

**DOI:** 10.1186/s13020-025-01281-0

**Published:** 2026-01-04

**Authors:** Kaixin Liu, Jin Yan, Fuyun Chi, Wenshuang Wang, Man Zhang, Yuanyuan Hou, Gang Bai

**Affiliations:** State Key Laboratory of Medicinal Chemical Biology, College of Pharmacy and Tianjin Key Laboratory of Molecular Drug Research, Nankai University, Tianjin, 300353 China

**Keywords:** Fuzheng Jiedu Formula, Acute pneumonia, NLRP3 inflammasome, Neutrophil NETosis, Host-directed therapies

## Abstract

**Background:**

Fuzheng Jiedu Formula (FZJD) is a polyherbal prescription that is commonly used in the treatment of infectious diseases, particularly infectious pneumonia. However, the key molecular mechanisms underlying its empirical therapeutic effects have not yet been fully elucidated. This study aimed to assess the anti-pneumonia efficacy of FZJD and elucidate its underlying regulatory mechanisms. Furthermore, it aimed to identify the plasma-exposed phytochemicals that may contribute to these pharmacological effects.

**Methods:**

A mouse model of *Pseudomonas aeruginosa* (PA-14) pneumonia was employed to evaluate the in vivo efficacy of FZJD. UPLC/Q-TOF-MS analyses were conducted to identify FZJD extracts and its plasma exposure components. Quantitative proteomics and non-targeted metabolomics combined with network pharmacology were used to map key molecular pathways. In parallel, in vitro assays conducted in macrophages and neutrophils evaluated the effects of FZJD’s key active compounds on inflammation and NETs formation.

**Results:**

Oral FZJD (10–40 g/kg) dose-dependently improved lung histopathology, limited macrophage and neutrophil infiltration, and lowered circulating TNF-α, IL-1β, and IL-6. Multi-omics integration analysis identified the NLRP3 signaling pathway and NETs formation as key dysregulated processes that were effectively reversed by FZJD treatment. Concordantly, lungs from treated mice showed lower p‑NF‑κB/NF‑κB, NLRP3, and cleaved caspase‑1, together with reduced cit‑H3/MPO and MPO-cfDNA complexes. Among 31 plasma-exposure constituents, saikosaponin A, prunasin, aloe-emodin, and glycyrrhizic acid emerged as multifunctional inhibitors that blocked NF-κB activation, curtailed NLRP3 assembly, and restrained NETosis in vitro. Pathway readouts supported actions on complementary axes (TLR4-IRAK1, STING1-IFN-β, FPR1-AKT, CASP8, and HDAC2-H3K9ac), providing a mechanistic basis for their collective protection against pneumonia.

**Conclusions:**

FZJD mitigates acute pneumonia by dampening macrophage NLRP3 inflammasome activation while restraining neutrophil NETosis. The mechanistic study provides evidence supporting the traditional use of FZJD in the treatment of respiratory infections and underscores its potential as a host-directed therapy.

**Graphical Abstract:**

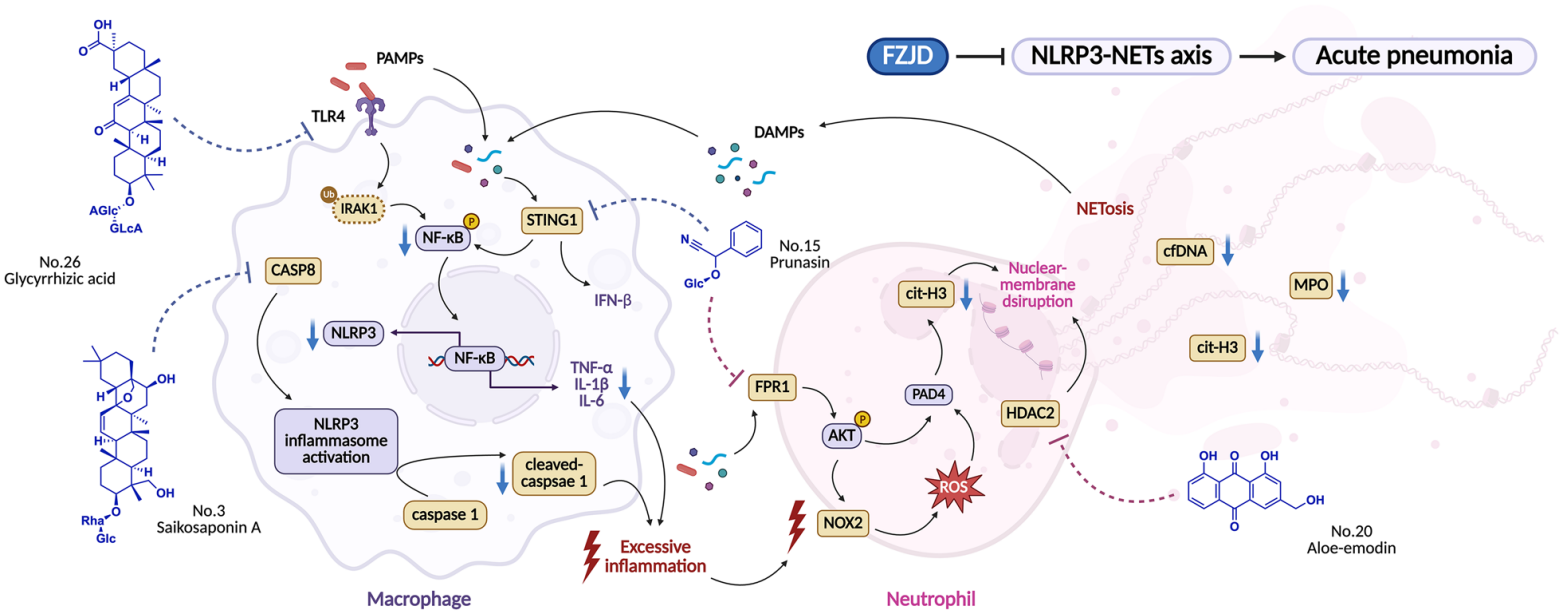

**Supplementary Information:**

The online version contains supplementary material available at 10.1186/s13020-025-01281-0.

## Background

Pneumonia is an acute infection of the alveoli and distal airways that may be caused by bacterial, viral or fungal pathogens [1]. Lower respiratory tract infections, of which pneumonia is a prominent example, caused an estimated 2.5 million deaths despite widespread access to antibiotics [2]. Clinical outcome is dictated not only by pathogen burden but also by the magnitude of the host inflammatory response, excessive cytokine release and alveolar damage often precipitate respiratory failure even after adequate antimicrobial therapy [3]. Consequently, adjunctive, host-directed therapies (HDTs) able to temper dysregulated immunity are urgently needed, especially in the face of rising antimicrobial resistance [4, 5].

Among the innate immune events that shape pneumonia progression, two inter-dependent processes stand out. The first is activation of the NOD-like receptor family pyrin domain-containing 3 (NLRP3) inflammasome in alveolar macrophages. Recognition of pathogen-associated molecular patterns (PAMPs) via toll-like receptors (TLRs) provides a “priming” signal that up-regulates pro-IL-1β and pro-IL-18, whereas a secondary danger signal drives NLRP3 assembly, caspase-1 cleavage and maturation of the two cytokines. Excessive production of IL-1β/IL-18 recruits further myeloid cells, amplifying the cytokine milieu and perpetuating tissue damage [6, 7]. The second process is the formation of neutrophil extracellular traps (NETs). NETs are web-like chromatin structures decorated with histones, myeloperoxidase (MPO) and neutrophil elastase. They are released primarily through NETosis, a specialized form of cell death that trap microorganisms but concurrently injure host tissue [8]. In experimental and clinical pneumonia, exuberant NETosis correlates with diffuse alveolar damage, impaired gas exchange and poor prognosis [9, 10]. Importantly, IL-1β and other NLRP3-derived mediators are potent NETs inducer, whereas NET-associated components such as cell-free DNA (cfDNA) and histones feedback to activate macrophages, driving further inflammasome activity [11–13]. Targeting either node in isolation has yielded limited clinical benefit: IL-1 antagonists blunt inflammation but do not prevent NET-driven barrier loss, whereas experimental NETs inhibitors fail when upstream inflammasome signaling remains uncontrolled [14, 15]. This underscores the need for dual-pathway modulators.

Traditional Chinese Medicine prescriptions, composed of multiple chemically diverse herbs, may intrinsically fulfill such polypharmacological requirements. For instance, Shiwei Longdanhua formula mitigates LPS-induced pulmonary inflammation and oxidative stress [16], Qingfei Yin suppresses necroptosis via autophagy activation in *Streptococcus pneumoniae* pneumonia [17], and Qingjin Huatan decoction alleviates influenza A virus pneumonia by modulating chemokine signaling [18]. However, most studies focus on the effects of whole formulas without specifically identifying bioactive constituents or systematically analyzing plasma exposure components, which results in discrepancies between in vitro bioactivity and in vivo relevance. The Fuzheng Jiedu Formula (FZJD) adopted in this study for the clinical treatment of infectious pneumonia from three classical formulations: Xiao Chai Hu Tang, Ma Xing Shi Gan Tang and San Ren Tang, all well-established in respiratory practice. The complete composition and botanical sources of FZJD are provided in Table S1. Each source formula contributes distinct therapeutic properties to pneumonia treatment. Ma Xing Shi Gan Tang has been widely used for severe respiratory infections, with both clinical and experimental studies demonstrating its effectiveness in reducing fever and alleviating inflammation [19, 20]. Key bioactive compounds, ephedrine, amygdalin and licoricesaponin G2, have been shown to exhibit anti-inflammatory, antitussive, antibacterial, and antiviral activities [21–23]. Xiao Chai Hu Tang has been evaluated in acute pneumonia and lower respiratory tract infections, where studies report improved symptom scores, shorter fever duration, and decreases in inflammatory markers [24, 25], underscoring its role in modulating systemic inflammatory responses. San Ren Tang, often incorporated into combination therapies for infectious pneumonia, demonstrates preclinical efficacy in attenuating airway inflammation and pulmonary edema. Clinical observational studies support its integration into treatment protocols for acute pneumonia [26, 27], highlighting its utility in mitigating pathological exudation and cellular infiltration. Together, these constituent formulars provide complementary mechanisms of action against pneumonia, which are strategically combined in FZJD, aligning traditional use with contemporary pharmacological evidence. Although individual components, such as baicalein from *Scutellaria baicalensis* Georgi [Lamiaceae] and resveratrol from *Polygonum cuspidatum* Siebold & Zucc. [Polygonaceae] have demonstrated anti-inflammatory and antioxidant effects in models of bacterial pneumonia [28, 29], the overall mechanism of action for the entire formula of FZJD remains to be elucidated.

In this study, we first investigated the therapeutic effects of FZJD on bacterial-induced infectious pneumonia through a systems pharmacology approach. After identifying the plasma exposure components and performing multi-omics analysis, we shifted our focus to the central macrophage-neutrophil interaction rather than individual inflammatory mediators. Leveraging insights from the cooperative dampening inhibitor, this study establishes a comprehensive HDTs mechanism for FZJD and outlines a methodological framework for researching other multi-target herbal medicines.

## Methods

### Preparation of FZJD

FZJD consists of 11 traditional Chinese medicinal herbs, all sourced from Tong Ren Tang (Beijing, China). Botanical identities were authenticated by prof. Min Jiang (Nankai University). Detailed preparation procedures and information of the drugs used were provided in Table S1.

### Chemicals and reagents

Levofloxacin (LVFX, S17134), saikosaponin A (B20146), saikosaponin B2 (B20148), saikosaponin C (B20149), saikosaponin D (B20150), amygdalin (B20687), emodin (B20240), aloe-emodin (B20772), glycyrrhizic acid (B20417), glycyrrhetic acid (S31512) (purity ≥ 98%), prunasin (B30413, purity ≥ 96%), polydatin (S31397, purity ≥ 95%), 2,4-decadienal (S45114, purity > 90%), were purchased from Yuanye (Shanghai, China). Lobetyolin (CFN99104) and tangshenoside I (CFN95108) (purity ≥ 98%) were obtained from Chemfaces. Ephedrine hydrochloride (171241) was sourced from National Institutes for Food and Drug Control (Beijing, China). Oroxindin (ST08350120), baicalein (ST01870120), wogonin (ST01710120), oroxylin A (ST23660120), resveratrol (ST00670120), coixol (ST80220120), cholesterol (ST03920120), apigenin (ST00410120), liquiritin (ST07010120), liquiritigenin (ST07000120) (purity ≥ 98%), and azelaic acid (STC2320200, purity ≥ 95%) were sourced from Nature Standard (Shanghai, China). 1,8-cineole (E809036, purity 99%) and Boc-MLF (B873861) were provided by Macklin (Shanghai, China). The antibodies for CD68 (28058-1-AP), MPO (22225-1-AP), p-NF-κB (Ser536, 80379-2-RR), NLRP3 (68102-1-Ig), interleukin-1 receptor-associated kinase 1 (IRAK1, 10478-2-AP) and β-actin (66009-1-Ig) were supplied by Proteintech (Wuhan, China). NIMP-R14 antibody (sc-59338) was obtained from Santa Cruz (Texas, USA). Antibodies against NF-κB (AF5006), caspase-1 (AF5418), cleaved caspase-1 (AF4005), AKT (AF6261), and p-AKT (AF0016) were acquired from Affinity (Jiangsu, China). Citrullinated histone H3 (cit-H3, ab5103) and MPO (ab300650) antibodies were obtained from Abcam (Cambridge, UK). Antibodies against H3K9ac (9649), caspase-8 (4790), cleaved caspase-8 (8592) were sourced from Cell Signaling Technology (Beverly, USA). TNF-α (021825) was sourced from PeproTech (New Jersey, USA). Pyrrolidine dithiocarbamate ammonium (PDTC, HY-18738), Cl-Amidine (HY-100574A), cycloheximide (CHX, HY-12320), TAK-242 (HY-11109) were acquired from Med Chem Express (New Jersey, USA). Z-IETD-FMK (KM5768), H-151 (KM3972), and N-Formyl-Met-Leu-Phe (fMLP, KM14705) were purchased from KKL Med (Virginia, USA). DMXAA (SC1031) and SAHA (SC0231) were sourced from Beyotime (Shanghai, China). LPS (L8880), and DAPI (C0065) were procured from Solarbio (Beijing, China).

### Cell culture

RAW 264.7 (CL-0190) and HEK 293T (CL-0005) cells (Procell, Wuhan, China) were maintained in DMEM supplemented with 10% FBS and 1 × penicillin/streptomycin at 37°C in 5% CO_2_. Experiments were performed at 70–80% confluence.

### Murine model of infectious pneumonia

Male KM mice (25 ± 2 g) were randomly assigned to six groups (*n* = 6): control (Con), model (Mod), positive control (LVFX, 60 mg/kg), and FZJD at 10, 20, and 40 g/kg (crud drug equivalent). Except for Con, mice received intranasal instillation of *Pseudomonas aeruginosa* PA-14 (20 μl, 1 × 10^7^ CFU/ml) and were held for 24 h to establish infection [30]. FZJD was administrated by oral gavage at -48 h and -24 h prior to inoculation, with additional doses given at the time of inoculation (0 h) and at + 8 h post-infection. LVFX was administrated by intraperitoneal injection at 0 h and 8 h post-inoculation. At 24 h post-infection, blood was collected, plasma prepared, and lungs were snap-frozen in liquid nitrogen and stored at -80°C for subsequent omics profiling. Additional lungs were fixed and paraffin-embedded for histology.

### Molecular networking and chemical scaffold classification

Male SD rats (250 ± 25 g) received oral FZJD (30 g/kg). Plasma was collected at 0.5, 1, 2, and 4 h via retro-orbital bleeding, extracted with methanol, and equal volumes from each time point were pooled. Pooled plasma and FZJD extract were analyzed by UPLC/Q-TOF-MS (Detailed in Method S1). MS/MS data were processed on GNPS (http://gnps.ucsd.edu), molecular networks were visualized in Cytoscape v3.10.1 [31], and chemical scaffolds were organized with Scaffold Hunter v2.6.3.

### Proteomics and metabolomics

Lung tissue samples and plasma from mice with infectious pneumonia were subjected to quantitative proteomics and non-targeted metabolomics analyses by Metware (Wuhan, China). Detailed protocols are provided in Method S2. Key affected pathways were identified via KEGG pathway enrichment (https://cn.string-db.org/).

### Network pharmacology

Structures of representative plasma-detected compounds were queried in SuperPred (https://prediction.charite.de/) to predict potential targets. Concurrently, target proteins associated with infectious pneumonia were retrieved from the GeneCards database (https://www.genecards.org), with only those scoring above 3 in relevance being retained. The intersection of targets from SuperPred with these high-relevance GeneCards targets was then used to identify potential infectious pneumonia-related targets modulated by FZJD.

### Histological and immunostaining analysis

Paraffin-embedded lungs were stained with hematoxylin and eosin (H&E) (G1120; Solarbio) to assess inflammatory infiltration. Histopathological scoring was determined according to previously reported methods [32]. Immunohistochemistry (IHC) was performed following the instructions provided with the IHC kit (PV-9000) and DAB chromogenic substrate solution (ZLI-9019; ZSGB-BIO, Beijing, China). This analysis aimed to detect CD68 and NIMP-R14 as markers of macrophage and neutrophil accumulation.

For immunofluorescence, sections were deparaffinized, antigen-retrieved, blocked (5% goat serum), incubated overnight at 4 °C with anti-cit-H3 (1:250) and anti-MPO (1:250), followed by a 1 h incubation at 25°C with Alexa Fluor® 488 and TRITC-conjugated species-specific secondary antibodies (1:750). Nuclei were counterstained with DAPI. Images were acquired on a TCS SP8 confocal microscope (Leica, Germany). NETs were identified by extracellular co-localization of cit-H3/MPO with decondensed DAPI-positive chromatin.

### NF-κB-luciferase activity assay

HEK 293T cells were plated in 96-well plates at 70% confluence and co-transfected for 24 h with 100 ng/well of the pGL4.32 luciferase reporter plasmid along with 10 ng/well of the Renilla luciferase reporter vector pRL-TK. Following transfection, the cells were treated with representative plasma exposure components at 1 μM, or with PDTC (10 μM) as positive controls. Among the 31 plasma-derived constituents, 27 were commercially available (≥ 95% purity) and therefore advanced to functional screening. All groups, except for the Con group, were then stimulated with 10 ng/ml TNF-α for 6 h. Firefly and Renilla luciferase activities were measured using a luciferase reporter gene detection system (E1980; Promega, Wisconsin, USA), with luminescence captured by a multifunctional microplate reader (Tecan, Austria), and expressed as firefly/Renilla ratios.

### In vitro immunofluorescence analysis of NLRP3 and NETs

RAW 264.7 cells were seeded on confocal dishes and assigned to Con, Mod, PDTC (10 μM), or treatment groups (1 μM). Except for Con, cells were exposed to LPS (1 μg/mL, 12 h), fixed (4% paraformaldehyde, 15 min), blocked, and incubated with anti‑NLRP3 (1:250, 4 °C, 12 h) followed by Alexa Fluor 488 secondary (1:750) and DAPI. For NETs, male C57BL/6J mice (25 ± 2 g) were euthanized and the femurs and tibias were dissected and processed to isolate bone marrow neutrophils following the protocol provided with the kit (P8550; Solarbio), the viability of freshly isolated neutrophils was determined by Trypan Blue exclusion (C0011S, beyotime) and was consistently > 90% before use in all downstream experiments. The neutrophils were plated on confocal dishes, pretreated for 1 h (Cl‑Amidine 1 μM as positive control or test compounds 1 μM), then stimulated with LPS (1 μg/mL, 4 h). Cells were stained with anti‑cit‑H3 (1:250) and anti‑MPO (1:250), followed by Alexa Fluor 488 (1:750) and TRITC (1:200), and DAPI. Imaging was performed on a TCS SP8 microscope.

### ELISA

RAW 264.7 macrophages were seeded in 96-well plates and assigned to Con, Mod, and treatment groups (*n* = 3). STING inhibitor H-151 (1 μM) served as the positive control. Selected representative plasma-exposed constituents (1 μM) were added for 1 h prior to stimulation. Cells were primed with LPS (1 μg/ml) for 4 h and then exposed to the murine STING agonist DMXAA (20 μM) for an additional 4 h. Supernatants were collected, and IFN-β levels were quantified using an ELISA kit (EK2236; Liankebio, Hangzhou, China) according to the manufacturer’s instructions.

Freshly isolated mouse neutrophils in 24-well plates were divided into Con, Mod, and treatment groups (*n* = 3). Peptidyl arginine deiminase 4 (PAD4) inhibitor Cl-Amidine (1 μM) was used as the positive control. After 1 h pretreatment, cells were stimulated with LPS (1 μg/mL, 4 h), and NETs were quantified as MPO-cfDNA complexes (JL47089; Jonlnbio, Shanghai, China).

Additionally, plasma samples from mice with infectious pneumonia were analyzed for the inflammatory cytokines TNF-α (JL10484), IL-1β (JL18442), and IL-6 (JLW20268), while lung homogenates were assessed for NETs measurement (JL47089). Absorbance at 450 nm was read and concentration calculated from standard curves.

### Western blot

Lung homogenates from PA‑14-infected mice (*n* = 5) were analyzed for NF‑κB, p‑NF‑κB, NLRP3, caspase‑1, cleaved caspase‑1, cit‑H3, and MPO.

In vitro assays were performed in 6-well plates (*n* = 3) with RAW 264.7 macrophages or primary neutrophils exposed to vehicle (Con), stimulus alone (Mod), positive control, or plasma-exposed constituents (1 μM). Stimulation paradigms and readouts were as follows: RAW 264.7—p‑NF‑κB/NF‑κB, NLRP3 (LPS 1 μg/mL, 12 h; PDTC 10 μM); RAW 264.7—cleaved caspase‑8/caspase‑8 (TNF-α 10 ng/mL + CHX 10 μg/mL, 4 h; Z‑IETD‑FMK 5 μM); RAW 264.7—IRAK1 (LPS 1 μg/mL, 15 min; TAK‑242 1 μM); neutrophils—cit‑H3/MPO (LPS 1 μg/mL, 4 h; Cl‑Amidine 1 μM); neutrophils—p‑AKT/AKT (fMLP 100 nM, 15 min; Boc‑MLF 1 μM); neutrophils—H3K9ac (LPS 1 μg/mL, 4 h; SAHA 1 μM).

Samples were lysed in RIPA with protease/phosphatase inhibitors, quantified, resolved by SDS‑PAGE, and transferred to PVDF membranes. After blocking, membranes were incubated with primary antibodies (typically 1:1000; β‑actin 1:20,000) and HRP‑conjugated secondary antibodies (1:10,000). Signals were captured on a Tanon‑5200 system and quantified in ImageJ. β‑actin was used as the loading control; phospho‑proteins were normalized to total protein.

### Statistical analysis

Statistical analysis was carried out using Graphpad Prism 9 software. Data are presented as means ± standard deviation. *t*-test was used to compare two groups, whereas one-way ANOVA was employed for comparisons among multiple groups. *p*-value < 0.05 was considered statistically significant.

## Results

### FZJD ameliorates infectious pneumonia in mice

**Fig. 1:**
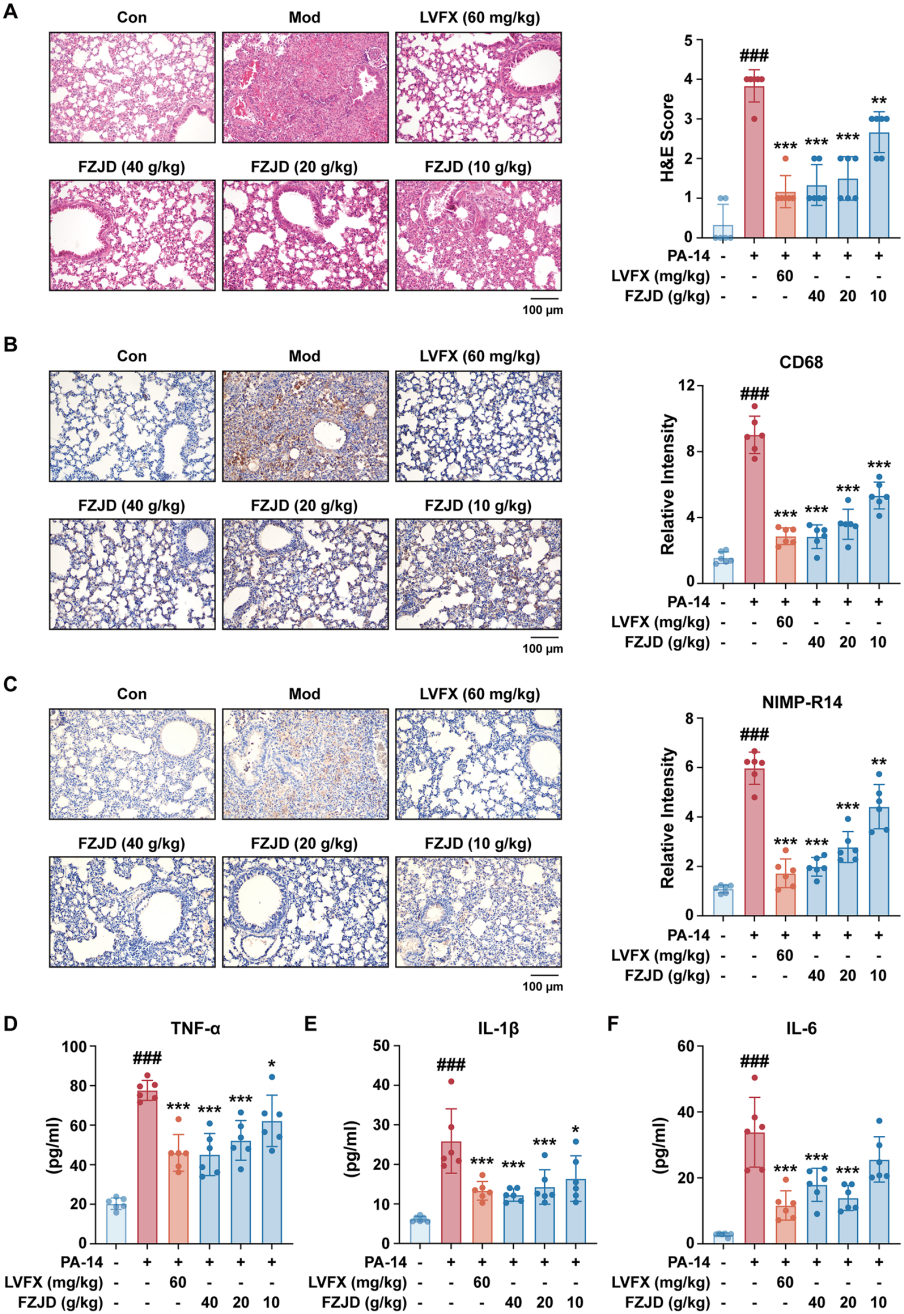
Fuzheng jiedu Formula (FZJD) alleviates lung pathology and attenuates inflammatory responses in mice with PA-14-induced infectious pneumonia. **A** Haematoxylin & eosin (H&E) and immunohistochemistry (IHC) staining of **B** CD68 and **C** NIMP-R14 in lung tissues. Plasma levels of pro-inflammatory cytokines **D** TNF-α, **E** IL-1β, and **F** IL-6 measured by ELISA. (levofloxacin (LVFX), 60 mg/kg, positive control; ###*p* < 0.001, *vs.* Con group; *p < 0.05, **p < 0.01, ***p < 0.001, *vs.* Mod group; *n* = 6)

Mice were intranasally inoculated with the *P. aeruginosa* PA-14 strain to assess the efficacy of FZJD in the treatment of pneumonia. As illustrated in Fig. 1A, H&E staining of lung sections demonstrated pronounced morphological damage in the Mod group, characterized by significant inflammatory cell infiltration and hemorrhage within the alveoli and airways. Treatment with low-, medium-, and high-dose FZJD (10, 20, and 40 g/kg, respectively) significantly alleviated the pathological changes in a dose-dependent manner, demonstrating progressively improved lung architecture compared to the Mod group. Further IHC staining revealed that FZJD markedly reduced macrophage accumulation, as evidenced by decreased CD68-positive regions in the lung tissue (Fig. 1B). Similarly, neutrophil marker NIMP-R14 staining demonstrated a significant reduction in neutrophil infiltration into the lung parenchyma (Fig. 1C). Additionally, plasma levels of pro-inflammatory cytokines TNF-α, IL-1β, and IL-6 were significantly elevated in the Mod group. However, FZJD administration markedly reduced these cytokine levels (Fig. 1D–F). These findings indicate that FZJD effectively alleviates pneumonia by reducing local and systemic inflammatory responses. In parallel, antibiotic LVFX (60 mg/kg) produced the expected amelioration of lung pathology and reductions in circulating cytokines, confirming that the PA-14 challenge is responsive to standard antibacterial therapy. Complementary data on 24 h survival, invasive lung mechanics, and intrapulmonary bacterial burden are provided in Fig. S1, showing that FZJD improves survival and lung function while only modestly reducing PA-14 signal, thereby supporting a primarily host-directed mode of action. Safety across the tested FZJD doses was also evaluated (Fig. S2).

### Identification of key components in FZJD

**Fig. 2:**
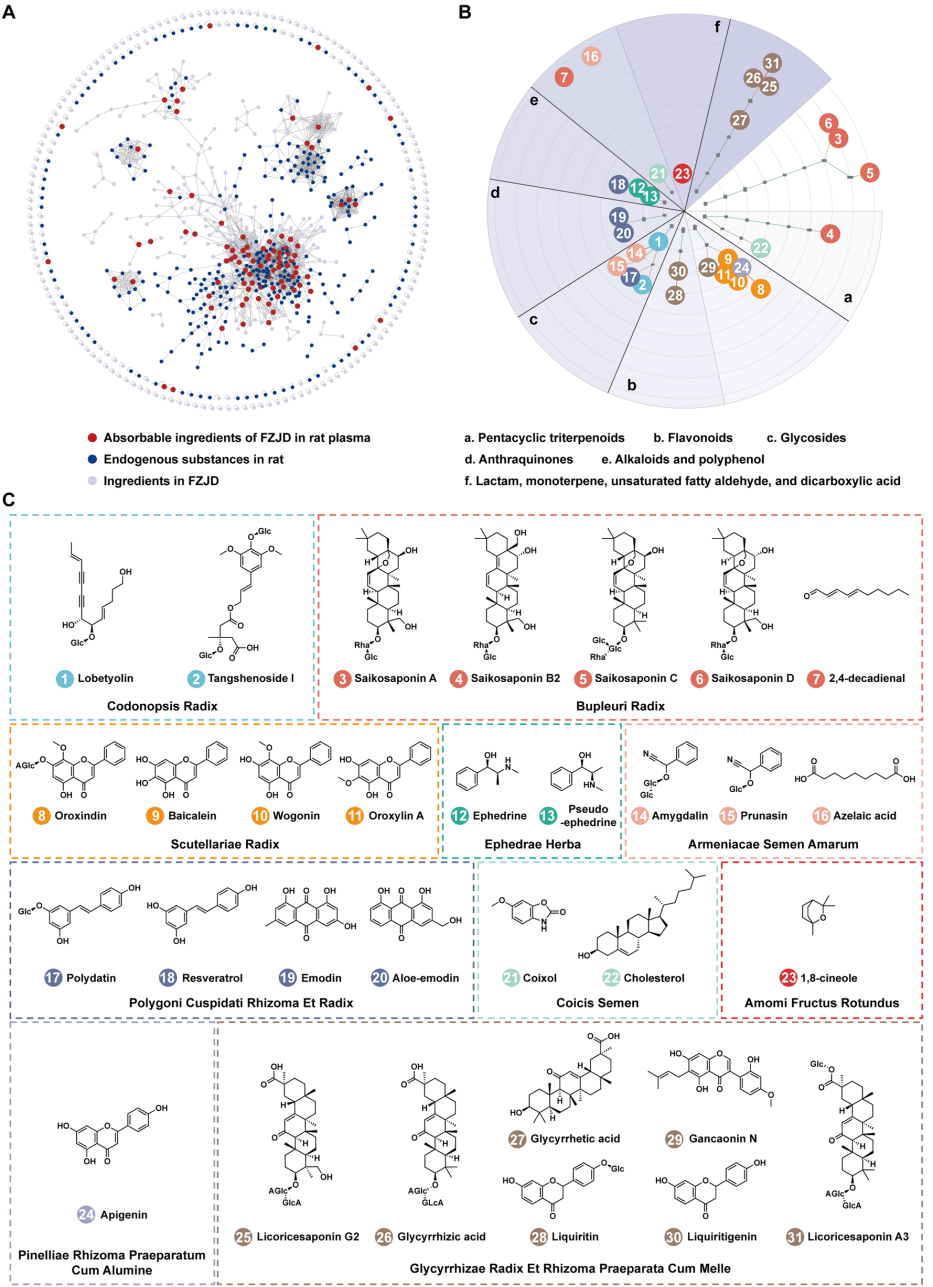
Systematic identification and characterization of FZJD ingredients. **A** Molecular network integrating extract, blank plasma, and post-dose plasma. **B** Scaffold-based chemotaxonomic classification, **C** structural annotation and crude drug sources for 31 plasma-exposed constituents

To identify potential ingredients absorbed into the blood following oral administration, UPLC/Q-TOF-MS analyses were conducted on FZJD extracts and plasma samples collected from rats at 0.5, 1, 2, and 4 h post-gavage. The acquired MS/MS data were then subjected to molecular networking analysis via GNPS. The resulting molecular network (Fig. 2A) revealed three distinct clusters: gray nodes representing the inherent ingredients of FZJD, blue nodes indicating the components of blank plasma, and 91 red nodes (intersection) corresponding to FZJD-derived plasma features.

Through systematic database matching (HMDB, PubChem, and MassBank) combined with accurate mass measurements and fragmentation pattern analysis, we identified 149 inherent chemical constituents in FZJD and 31 prototype plasma exposure constituents (Fig. S3, S4; Table S2). Scaffold classification of these 31 ingredients using Scaffold Hunter revealed their structural diversity (Fig. 2B): 9 pentacyclic triterpenoids (cluster a), 8 flavonoids (cluster b), 5 glycosides (cluster c), 2 anthraquinones (cluster d), 2 alkaloids and 1 polyphenol (cluster e), 1 lactam, 1 monoterpene, 1 unsaturated fatty aldehyde, and 1 dicarboxylic acid (cluster f). The chemical structures of these ingredients are illustrated in Fig. 2C, along with their corresponding crude drug sources. These 31 plasma-exposed constituents were subsequently prioritized for network pharmacology analysis and mechanistic validation.

### Integrated proteo-metabolomics pinpoints NOD-like receptor signaling and NETs formation as core pathways modulated by FZJD

**Fig. 3:**
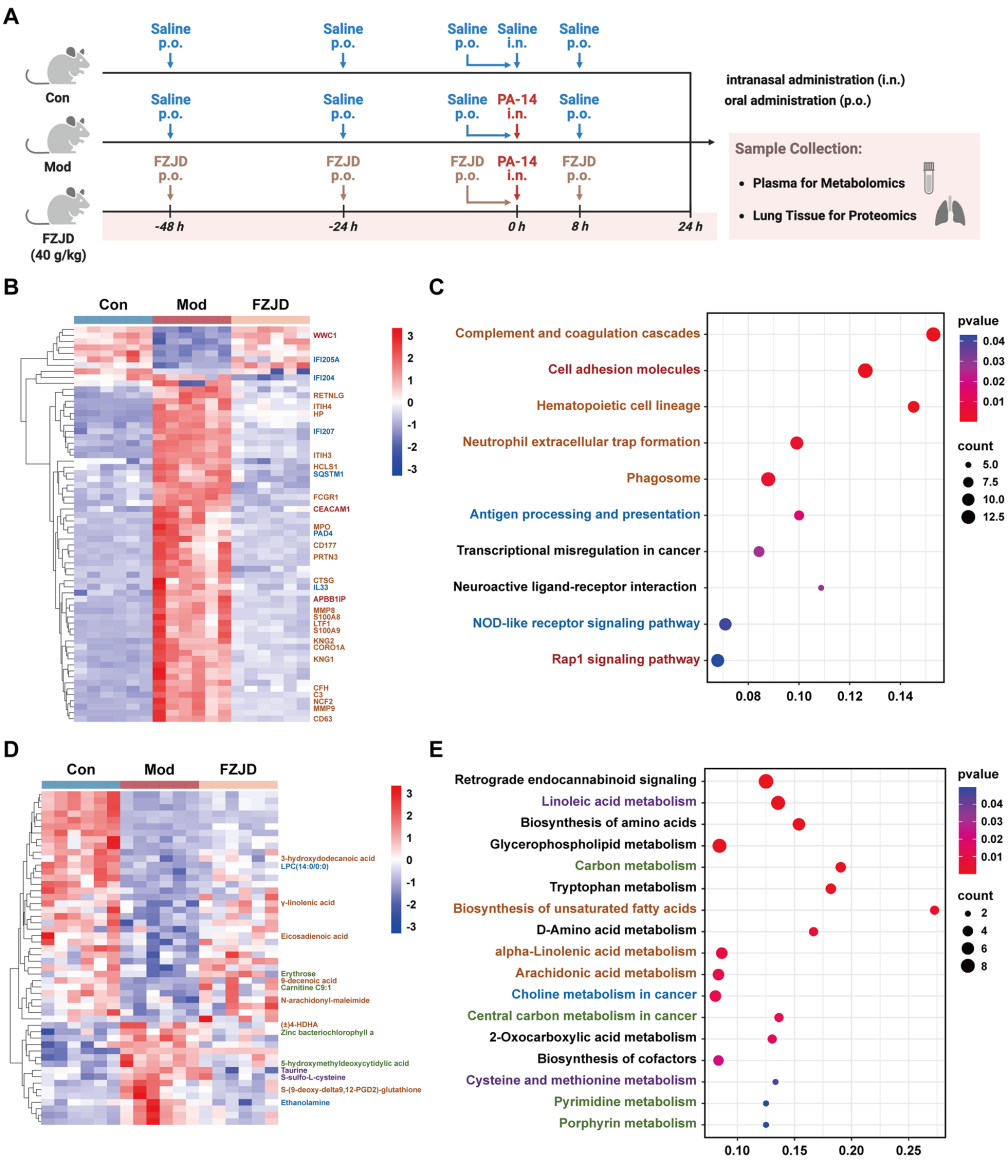
Multi-omics profiling reveals FZJD-mediated regulation of infectious pneumonia. **A** Mice experimental workflow for proteomic and metabolomic analyses. **B** Normalized heatmap and **C** KEGG pathway enrichment of 1062 reversed differentially expressed proteins (DEPs) in lung tissues used for proteomic analysis. **D** Heatmap and **E** KEGG pathway enrichment of 395 reversed differentially expressed metabolites (DEMs) in plasma used for metabolomic analysis

To decipher the mechanism underlying FZJD's anti-pneumonia effects, we performed quantitative proteomics and non-targeted metabolomics on lung tissue and plasma samples from the Con, Mod, and FZJD-treated (40 g/kg) groups. The experimental workflow was illustrated in Fig. 3A.

Proteomics identified a total of 9284 proteins, among which 1062 differentially expressed proteins (DEPs) exhibited significant reversal of Mod-induced dysregulation in the FZJD group (Fig. 3B, Fig. S5A). KEGG pathway enrichment analysis of these DEPs highlighted several critical pathways (Fig. 3C). During this process, innate immunity was activated, resulting in the formation of the complement and coagulation cascades, phagosome, hematopoietic cell lineage and NETs, all of which are essential for bacterial clearance [33–36]. To aid interpretation, the heatmap labels representative proteins within these nodes, including complement/kinin factors (C3, CFH, KNG1/KNG2, ITIH3/ITIH4, HP), phagosomal and NET-associated enzymes (NCF2, FCGR1, CD63, CORO1A, MPO, CTSG, PRTN3, MMP8/MMP9, S100A8/S100A9, LTF), and markers of granulocytic mobilization (CD177, CEACAM1, RETNLG, HCLS1). Meanwhile, the inhibition of cell adhesion molecules and the Rap1 signaling pathway suggested effective regulation of immune cell migration, consistent with the annotation of APBB1IP, CEACAM1 and WWC1 [37, 38]. Antigen processing and presentation activated specific immune responses to help eliminate bacteria and form immune memory [39]. Additionally, the activation of NLRP3 mediated by the NOD-like receptor signaling pathway was also modulated, as reflected by the annotated sensors and amplifiers IFI204/IFI205A/IFI207, IL-33, SQSTM1 and the NET-execution factor PAD4 [7].

Metabolomic analysis identified 2413 metabolites, of which 395 differentially expressed metabolites (DEMs) in the FZJD group mitigated Mod-induced metabolic perturbations (Fig. 3D, Fig.S5B). KEGG enrichment analysis revealed that the upregulation of energy metabolic pathways, including carbon metabolism, central carbon metabolism, pyrimidine metabolism and porphyrin metabolism provided essential energy for immune cell activation (Fig. 3E) [40–43]. In support, the metabolite C9:1 (central carbon/β‑oxidation), 5-hydroxymethyldeoxycytidylic (pyrimidine) and zinc bacteriochlorophyll a (porphyrin). Alterations in arachidonic acid metabolism, alpha-linolenic acid metabolism, and biosynthesis of unsaturated fatty acids indicated controlled inflammation progression [44, 45]. In support, the metabolite heatmap highlights γ‑linolenic acid, eicosadienoic acid, N-arachidonyl-maleimide, ( ±)4‑HDHA, and 9-decenoic acid, which together trace the realignment of pro- and anti-inflammatory lipid mediators. Furthermore, the observed changes in cysteine and methionine metabolism and linoleic acid metabolism suggest a potential reduction in oxidative stress [46, 47]. This is reflected by the annotation of S-sulfo-L-cysteine and taurine, both implicated in reactive species buffering. The involvement of choline metabolism in cell membrane synthesis indicated an enhancement in cell membrane integrity, consistent with the labelled LPC(14:0/0:0) and ethanolamine that point to phospholipid turnover and repair [48].

**Fig. 4:**
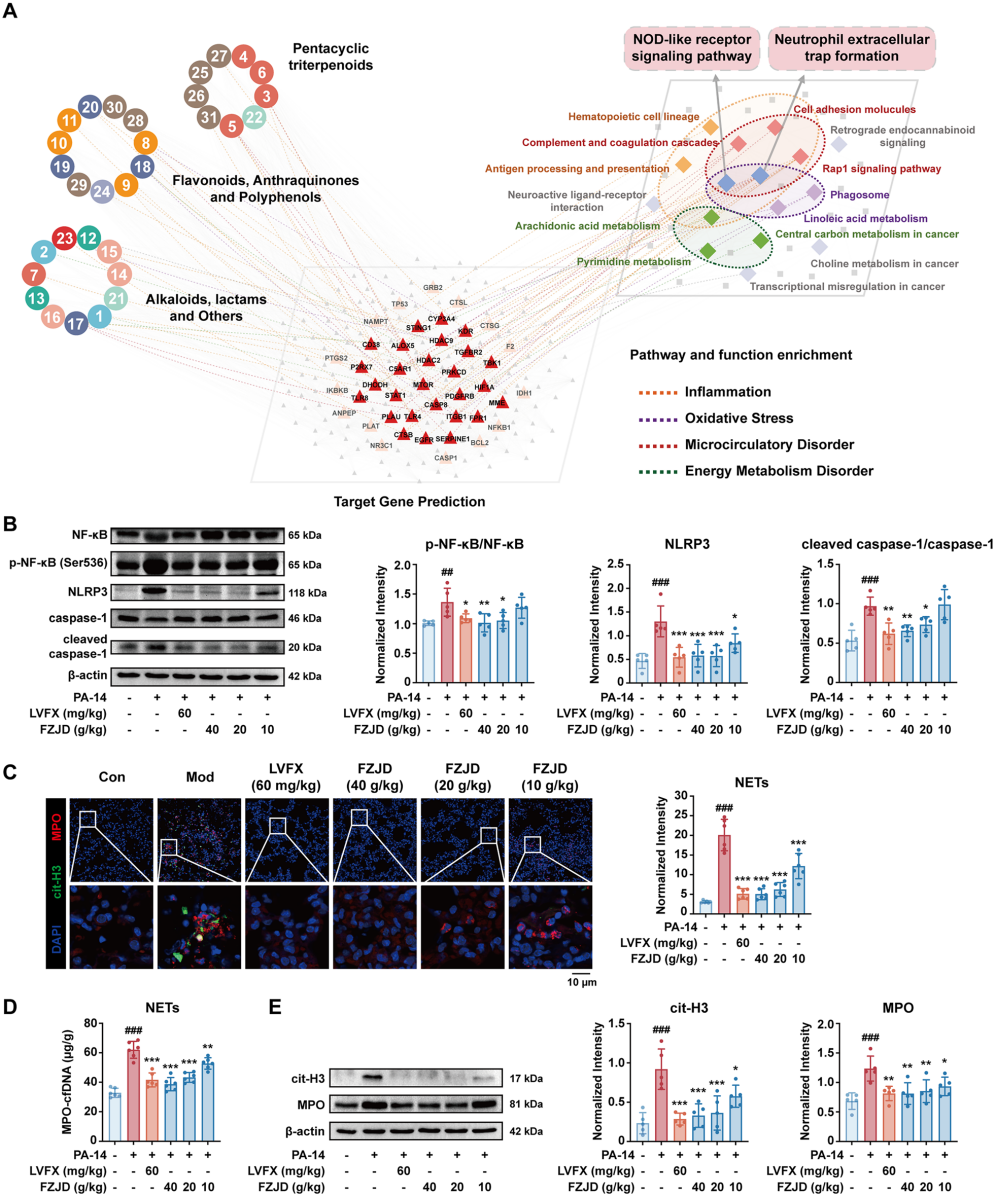
Integration analysis reveals inhibition of NOD-like receptor signaling and NETs formation. **A** Integrated functional landscape network of FZJD. Layer 1: 31 plasma-exposed ingredients, grouped by scaffold class; layer 2: predicted protein targets (SuperPred) filtered by pneumonia relevance (GeneCards score > 3); layer 3: pathways/biological functions significantly enriched by reversed DEPs (lung proteomics) and DEMs (plasma metabolomics) (KEGG). Edges from ingredients to targets denote predicted interactions; edges from targets with KEGG pathways supported by our omics enrichment. **B** Western blots of NF-κB, p-NF-κB, NOD-like receptor family pyrin domain containing 3 (NLRP3), caspase-1, and cleaved caspase-1 in lung homogenates (β-actin loading control; *n* = 5). **C** Lung immunofluorescence for neutrophil extracellular traps (NETs) (citrullinated histone H3 (cit-H3), myeloperoxidase (MPO), DAPI; *n* = 6). **D** ELISA of NETs (MPO-cfDNA complexes) in lung homogenates (*n* = 6). **E** Western blots of cit-H3 and MPO in lung homogenates (β-actin loading control; *n* = 5). (##*p* < 0.01, ###*p* < 0.001, *vs.* Con group; *p < 0.05, **p < 0.01, ***p < 0.001, *vs.* Mod group)

To establish a connection between the chemical exposure component and system-level biological effects, we constructed an integrated network encompassing constituents, molecular targets, and signaling pathways. We firstly compiled a “pathway pool” from KEGG terms significantly enriched by FZJD-reversed DEPs in lung and DEMs in plasma (Fig. 3C, E). The 31 plasma-detected constituents were subjected to target prediction using SuperPred to identify putative human targets, which were subsequently converted to their mouse orthologues. These predicted targets were then intersected with high-relevance infectious pneumonia-associated targets retrieved from GeneCards. Then the retained targets were subsequently mapped back to the pathway pool, and pathways containing multiple supported targets were clustered into distinct functional modules. The final tripartite network (Fig. 4A) illustrated: Layer 1, comprising 31 exposed constituents categorized by scaffold class; Layer 2, encompassing shared targets identified through predictive assay; and Layer 3, representing convergent pathways aggregated into four functional modules (anti-inflammation, anti-oxidant, microcirculatory regulation, and energy metabolism). Within the integrated framework, NOD-like receptor signaling and NETs formation emerge as highly connected hubs, suggesting a coordinated suppression of macrophage inflammasome activation and neutrophil NETosis by FZJD.

We then validated these two routes in vivo. Western blotting showed suppression of upstream inflammatory signaling in FZJD-treated lungs, including a reduced p-NF-κB/NF-κB ratio, and decreased NLRP3 and cleaved caspase-1/caspase-1 relative to the Mod group (Fig. 4B; Fig. S6, S7) Immunofluorescence revealed abundant cit-H3/MPO-positive web-like structures in Mod lungs that were markedly diminished by FZJD (Fig. 4C). Consistently, MPO-cfDNA complexes in lung homogenates were reduced compared with the Mod (Fig. 4D). Western blot further confirmed reduced cit-H3 and MPO (Figs 4E; Fig. S8). Together, these data indicate that FZJD mitigates pneumonia predominantly through dampening NLRP3 inflammasome activation and curbing NETs formation in the lung tissue.

### Activity-guided validation of NOD-like receptor signaling modulators

**Fig. 5:**
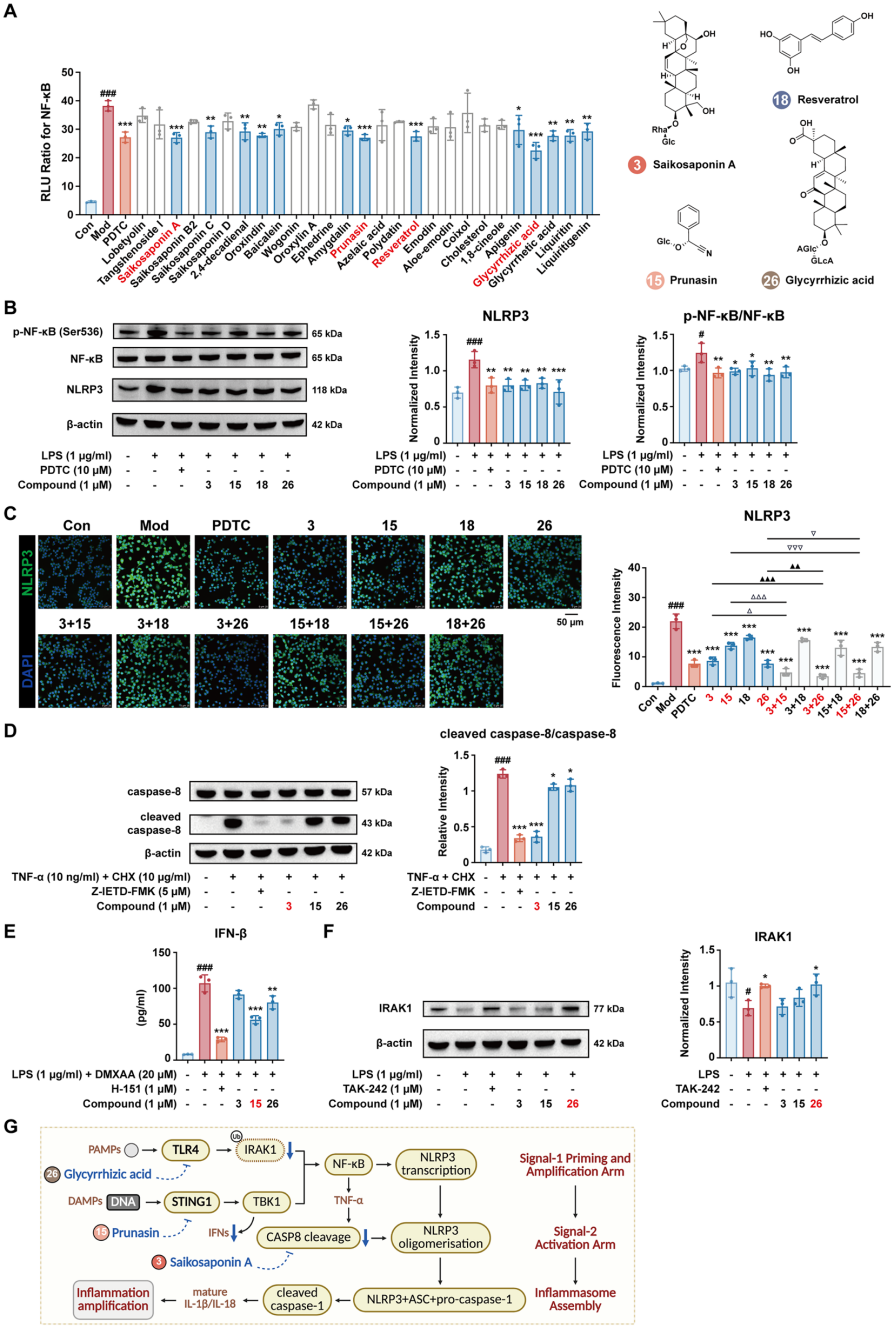
Screening and validation of plasma-exposed constituents that modulate the NOD-like receptor signaling. **A** NF-κB inhibition via luciferase reporter assays in HEK-293T cells. **B** Western blot of p-NF‑κB (Ser 536), NF‑κB, NLRP3 in LPS-stimulated RAW 264.7 cells (β-actin loading control). **C** NLRP3 (green) immunofluorescence showing monomer and pairwise combination effects. **D** Western blot of caspase-8 and cleaved caspase-8 in RAW 264.7 cells stimulated with TNF-α + CHX (Z-IETD-FMK, 5 μM, positive control; β-actin loading control). **E** IFN-β ELISA in RAW 264.7 cells under LPS priming + DMXAA (H-151, 1 μM, positive control). **F** Western blot of interleukin-1 receptor-associated kinase 1 (IRAK1) in LPS-stimulated RAW 264.7 cells (TAK-242, 1 μM, positive control; β-actin loading control). **G**. Multi-node restraint of NLRP3 signaling by three FZJD constituents. (#*p* < 0.05, ###*p* < 0.001, *vs.* Con group; *p < 0.05, **p < 0.01, ***p < 0.001, *vs.* Mod group; △p < 0.05, △△△p < 0.001, ▲▲p < 0.01, ▲▲▲p < 0.001, ▽p < 0.05, ▽▽▽p < 0.001, vs. monomers corresponded groups; *n* = 3)

We initially screened 27 available plasma-derived constituents (1 µM) for NF-κB inhibitory activity using a luciferase reporter assay in HEK-293T cells. At this concentration (1 µM), no effect on cell viability was observed (Fig. S9). Four compounds, saikosaponin A (No. 3), prunasin (No. 15), resveratrol (No. 18), and glycyrrhizic acid (No. 26), demonstrated the most significant suppression of TNF-α-induced NF-κB activity, mirroring the inhibition achieved with the pathway inhibitor PDTC (*p* < 0.001; Fig. 5A). In LPS-stimulated RAW 264.7 macrophages, all four compounds markedly reduced NLRP3 abundance and reduced the p-NF‑κB/NF‑κB ratio, PDTC (10 µM) likewise dampened this NF-κB-dependent priming step, supporting specificity of the readout (Fig. 5B; Fig. S10). Immunofluorescence analysis confirmed reduced NLRP3 staining. Furthermore, pairwise combinations (saikosaponin A + prunasin; saikosaponin A + glycyrrhizic acid; prunasin + glycyrrhizic acid) exhibited stronger NLRP3 suppression than individual treatments, demonstrating a cooperative inhibitory effect (Fig. 5C).

To validate the pathway-level associations predicted by network pharmacology that saikosaponin A interacts with CASP8, prunasin with STING1, and glycyrrhizic acid with TLR4 (Fig. S11A-C), we experimentally verified the changes in downstream molecules. Consistent with network-guided assignments, saikosaponin A decreased caspase-8 activation in the TNF-α + CHX paradigm, in line with the caspase-8 inhibitor Z-IETD-FMK used as an activity control for this cleavage readout (Figs. 5D; S12); Prunasin significantly lowered IFN-β release upon DMXAA stimulation (with LPS priming) RAW 264.7 cells, matching the effect of the STING inhibitor H-151 and confirming pathway dependence (Fig. 5E). Additionally, TAK-242, which binds the intracellular domain of TLR4, stabilized IRAK1 protein after LPS exposure, and glycyrrhizic acid produced the same rescue (Fig. 5F; Fig. S13). Collectively, these data support multi-node restraint of the NF-κB/NLRP3 axis by screen-selected constituents.

To contextualize these readouts, canonical NLRP3 activation proceeds in two steps. Priming (signal-1) via TLR ligands or type I interferons induces NLRP3 and pro-IL-1β transcription through NF-κB/STATs; activation (signal-2) is triggered by danger cues (K^+^ efflux, ROS, lysosomal stress), driving NLRP3 oligomerization with ASC and pro-caspase-1. Caspase-8 can substitute for or cooperate with caspase-1 in this second step, lowering the activation threshold. By attenuating TLR4-IRAK1 (glycyrrhizic acid), STING1-dependent type I IFN (prunasin), and caspase-8 cleavage (saikosaponin A), the three constituents intercept both priming and activation, collapsing inflammasome assembly and downstream IL-1β/IL-18 maturation (Fig. 5G).

### Activity-guided validation of NETs formation modulators

**Fig. 6:**
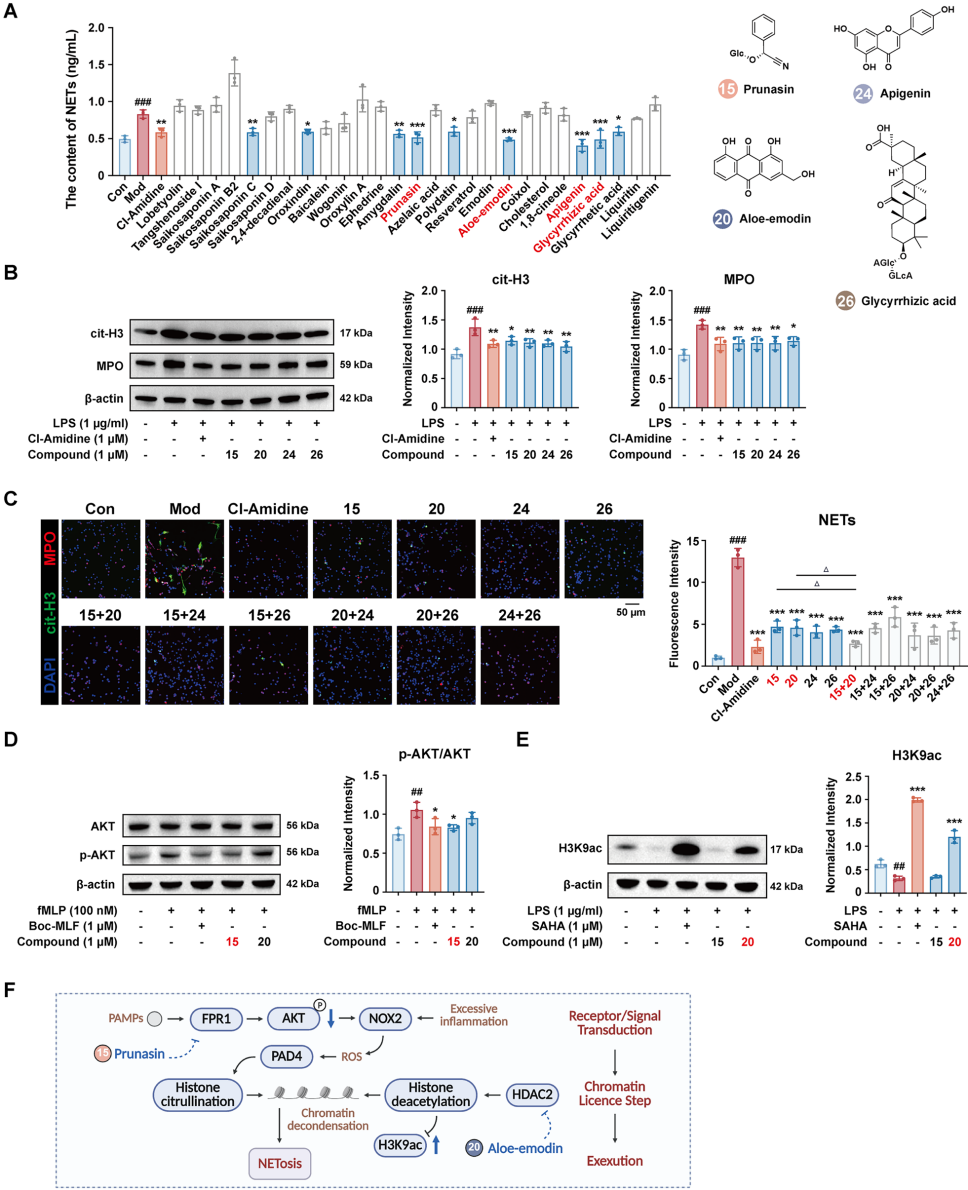
Screening and validation of plasma-exposed constituents that modulate the NETs formation. **A** NETs ELISA (MPO-cfDNA) screen and **B** Western blot of cit-H3 and MPO in LPS-stimulated neutrophils (β-actin loading control). **C** Immunofluorescence of cit-H3 (green)/MPO (red)/DAPI (blue) showing monomers and pairwise combination effects. **D** Western blot of p-AKT/AKT in N-Formyl-Met-Leu-Phe (fMLP)-stimulated neutrophils (Boc-MLF, 1 μM, positive control; β-actin loading control). **E** Western blot of H3K9ac in LPS-stimulated neutrophils (SAHA, 1 μM, positive control; β-actin loading control). **F** Dual blockade of NETs formation by two FZJD constituents. (##*p* < 0.01, ###*p* < 0.001, *vs.* Con group; *p < 0.05, **p < 0.01, ***p < 0.001, *vs.* Mod group; △p < 0.05, vs. monomers corresponded groups; *n* = 3)

In primary mouse neutrophils, we screened the same 27 plasma-exposed constituents (1 μM) for NETs suppression, with the PAD4 inhibitor Cl-Amidine (1 μM) included as a mechanistic control. Prunasin (No. 15), aloe-emodin (No. 20), apigenin (No. 24), and glycyrrhizic acid (No. 26) stood out and reduced LPS-induced NETs quantified as MPO-cfDNA complexes (*p* < 0.001), and Cl-Amidine produced a comparable decrease, consistent with PAD4-depedent chromatin decondensation in this assay (Fig. 6A). Western blotting also corroborated lower cit-H3 and MPO levels across all four compounds and Cl-Amidine (Fig. 6B; Fig. S14). Noteworthy, immunofluorescence confirmed fewer cit-H3/MPO-positive web-like structures with the prunasin + aloe-emodin combination showing the greatest attenuation (Fig. 6C).

Subsequently, the synergistic inhibitory effect was clarified at the downstream pathway level by the guidance provided by the prediction results from network pharmacology, which prunasin target with FPR1, and aloe-emodin target with HDAC2 (Fig. S11D, E). As expected, Boc-MLF, an FPR1 antagonist, blunted fMLP-evoked AKT phosphorylation, while prunasin produced an identical attenuation (Fig. 6D; Fig. S15). Conversely, the pan-HDAC inhibitor SAHA restored H3K9 acetylation under LPS challenge, while aloe-emodin raised H3K9ac to a similar extent (Fig. 6E; Fig. S16). These findings indicate complementary signaling (FPR1-AKT) and chromatin (HDAC2-H3K9ac) routes converging to restrain NETs formation.

NETs formation is likewise staged. Surface receptors such as FPR1 or TLRs trigger PI3K-AKT and NADPH oxidase, generating ROS that license PAD4-mediated histone citrullination. In parallel, chromatin must achieve a permissive acetylation state (regulated in part by HDAC2) before large-scale decondensation. Our data place prunasin upstream, limiting FPR1-AKT signaling, and aloe-emodin downstream, inhibiting HDAC2 and restoring H3K9ac. This dual intervention converges on reduced cit-H3/MPO release and lower NETs output, rationalizing the additive-to-synergistic effect of the combination (Fig. 6F).

## Discussion

Severe pneumonia often deteriorates after pathogen titres plateau, indicating that dysregulated immunity, not residual pathogen load, precipitates respiratory failure [49]. Two innate programmes dominate this maladaptation. Activation of the NLRP3 inflammasome in alveolar macrophages produces mature IL-1β and IL-18, which recruit and prime neutrophils. Primed neutrophil, in turn, releases NETs—chromatin webs decorated with histones, MPO and elastase—that exacerbate epithelial damage and serve as damage-associated molecular patterns (DAMPs) to reactive macrophage inflammasomes [13, 50]. This vicious cycle has been described in bacterial, viral and aspiration pneumonias [51–53], underscoring its translational relevance. In our model, omics integration pinpointed NOD-like receptor signaling and NETs formation as the two most significantly reversed pathways, and histological as well as biochemical readouts confirmed the events in vivo.

**Fig. 7:**
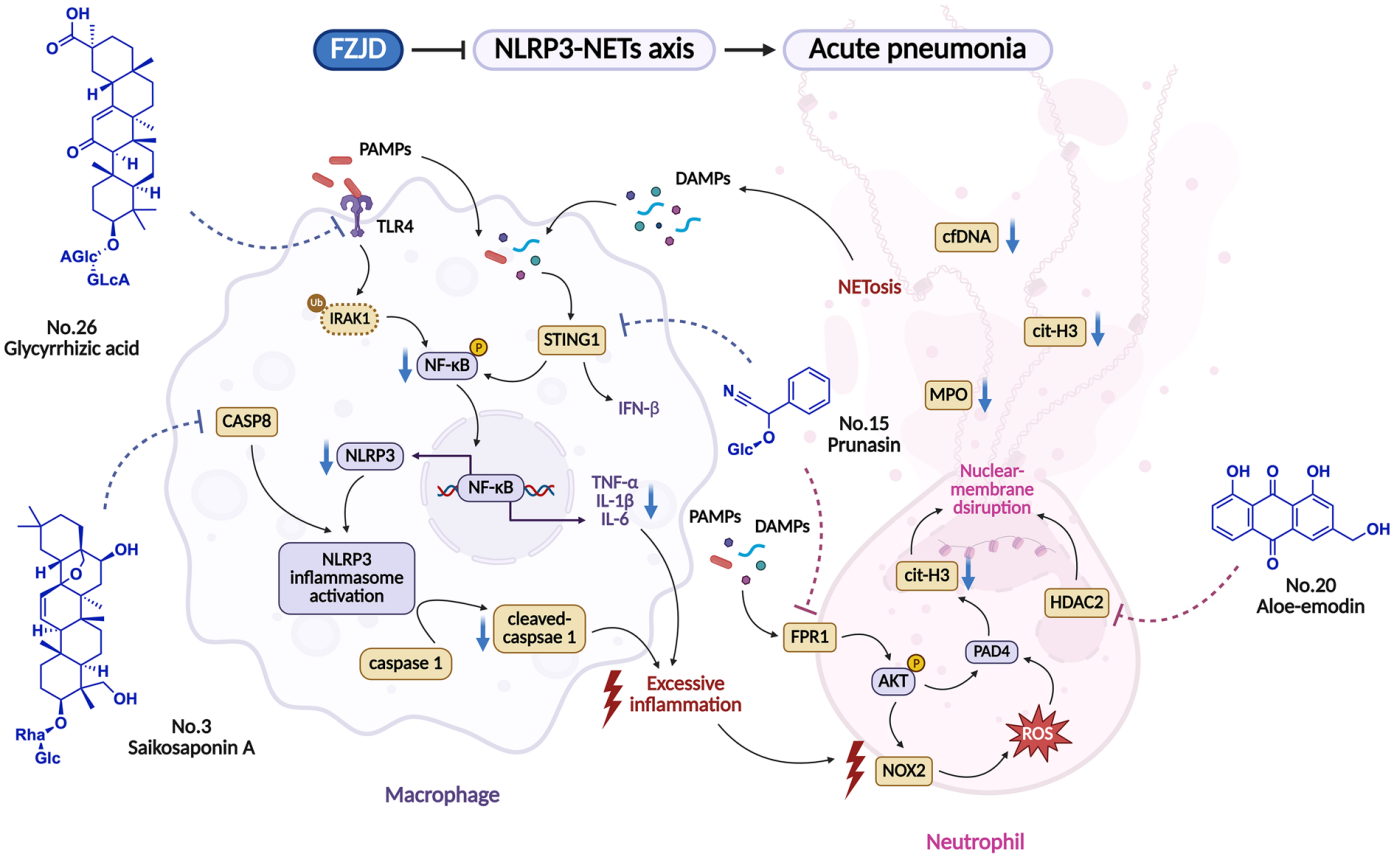
The mechanism diagram illustrates that FZJD ameliorates acute pneumonia

Because the NLRP3-NETs circuit involves at least two cell types and several sequential steps (pathogen sensing, inflammasome priming, caspase-1 cleavage, chromatin decondensation), single-target interventions have produced inconsistent clinical benefit. IL-1 antagonists attenuate cytokinaemia but fail to prevent NETs-mediated barrier loss. Conversely, PAD4 inhibitors reduce NETosis yet leave upstream IL-1β unchecked, providing at best transient functional improvement [14, 15]. However, our data demonstrated that multi-ingredient preparation can overcome this limitation. Prototype or metabolic components derived from FZJD construct a cooperative ensemble that is required to recurring the synergistic effects observed in vivo (Fig. 7). By reducing NF-κB-mediated priming, blocking NLRP3 inflammasome assembly, and directly inhibiting NETosis, the formula effectively removed the inflammatory cascade, thereby leading to consistent reductions in alveolar consolidation, myeloid cell infiltration, and systemic cytokine levels.

A persistent challenge in ethnopharmacology is linking the multitude of herbal constituents to tangible in vivo actions. We addressed this with an exposure-guided pipeline: UPLC/Q-TOF-MS and molecular networking narrowed 149 extractable molecules to 31 circulating constituents; among these, 27 accessible compounds were screened in unbiased assays. Analysis of TNF-α-induced NF-κB reporter activity in HEK-293T cells and LPS-stimulated NET formation in neutrophils identified four key constituents and their respective node-specific signaling pathways: glycyrrhizic acid (TLR4-IRAK1), prunasin (STING1-IFN-β; FPR1-p-AKT/AKT), saikosaponin A (CASP8-cleaved/total caspase-8), and aloe-emodin (HDAC2-H3K9ac). These nodes are, TLR4 and FPR1 provide surface sensing and NF‑κB/PI3K‑AKT entry [54, 55], STING1 couples cytosolic nucleic‑acid surveillance to type I IFN [56], CASP8 integrates extrinsic apoptotic signaling with NLRP3 activation [57], and HDAC2 governs histone acetylation and chromatin readiness for NETs release [58]. Their combined modulation plausibly maps onto reduced NLRP3 signaling and NETs formation. The same exposure-anchored, omics-guided pipeline can be generalized to other multi-herb formulas to convert chemical complexity into a tractable, mechanism-based narrative.

External evidence also supports this placement. Saikosaponin A suppresses NF-κB and NLRP3 and alleviates LPS-induced lung injury [59]; glycyrrhizic acid dampens HMGB1/TLR4-dependent NLRP3 activation [60, 61]; and amygdalin, as a prototype component of prunasin, lowers TNF-α, IL-1, and IL-6 and suppresses NF-κB/MAPK signaling [62], consistent with our observation that prunasin restrains the STING1-IFN‑β arm in macrophages and the FPR1-AKT axis in neutrophils. For aloe-emodin, reported HDAC inhibition aligns with our HDAC2-centric readouts: restoration of H3K9ac under inflammatory stimulation and reduced NETs output [63]. Independent studies likewise link lower p-AKT to NETs suppression, reinforcing these mechanistic assignments [64, 65]. By acting at complementary entry points, these constituents blunt priming (TLR4/NF-κB), signal-2 integration (CASP8), and execution (HDAC2-dependent chromatin decondensation) within the NLRP3-NETs circuit, providing a coherent rationale for the cooperative dampening of early cytokine surges, neutrophil recruitment, and NETosis observed with FZJD.

In clinical adjunctive corticosteroids, IL-6 receptor antagonists and JAK inhibitors can improve oxygenation in select pneumonia cohorts, but they also increase secondary infections and have not consistently reduced mortality [66, 67]. However, HDTs aim to recalibrate rather than suppress immunity and are therefore considered safer and more durable [68]. FZJD targets multiple host pathways that are central to tissue damage and demonstrates therapeutic efficacy independent of bacterial killing. Its polypharmacological approach further minimizes the risk of developing pharmacological resistance, a major limitation associated with monoclonal antibodies and single-kinase inhibitors. These characteristics position FZJD as a promising adjunct to antibiotics, especially in the context of increasing antimicrobial resistance.

While the present study integrates in vivo pharmacology, multi-omics, and exposure-guided validation, limitations should be noted. This study employed only male mice. Given documented sexual dimorphism in innate immune responses, including macrophage inflammasome signaling and neutrophil NETosis [69, 70], future investigations will incorporate female cohorts to validate the macrophage NLRP3 and neutrophil NETs outcomes and to evaluate potential sex-specific pharmacodynamic effects of FZJD. Secondly, the PA-14 mouse model captures an acute Gram-negative pneumonia over a short window (24 h), whether similar protection extends to other pathogens or chronic models remains to be determined. Additionally, pathway readouts (IRAK1 degradation, IFN‑β release, cleaved/total caspase‑8, p‑AKT/AKT, H3K9ac) are informative but not target-exclusive. These issues are addressable in future studies and do not detract from the overall mechanistic coherence of the findings.

## Conclusions

In summary, dismantling the NLRP3-NETs feedback loop represents a promising therapeutic strategy for severe pneumonia, and FZJD provides a clinically derived polypharmacological approach to target these mechanisms. This study bridges traditional ethnomedicine with modern immunology, highlighting the potential of FZJD in advancing HDTs for complex infectious diseases, thereby addressing urgent medical needs in the context of rising antimicrobial resistance.

## Supplementary Information

Additional file 1.

## Data Availability

The data that support the findings of this study are available from the corresponding author upon reasonable request.

## Abbreviations

HDTs: Host-directed therapies
NLRP3: NOD-like receptor family pyrin domain containing 3
PAMPs: Pathogen-associated molecular patterns
TLRs: Toll-like receptors
NETs: Neutrophil extracellular traps
MPO: Myeloperoxidase
cfDNA: Cell-free DNA
FZJD: Fuzheng Jiedu Formula
LVFX: Levofloxacin
IRAK1: Interleukin-1 receptor-associated kinase 1
cit-H3: Citrullinated histone H3
PDTC: Pyrrolidine dithiocarbamate ammonium
CHX: Cycloheximide
fMLP: N-Formyl-Met-Leu-Phe
Con: Control
Mod: Model
H&E: Hematoxylin and eosin
IHC: Immunohistochemistry
PAD4: Peptidyl arginine deiminase 4
DEPs: Differentially expressed proteins
DEMs: Differentially expressed metabolites
DAMPs: Damage-associated molecular patterns
