# Supplementary material for: Fuzheng Jiedu formula attenuates acute pneumonia by coordinated regulation of macrophage NLRP3 inflammasome and neutrophil NETs
Source: Chin Med. 2026 Jan 4;21:1. doi: 10.1186/s13020-025-01281-0 (PMC12765288; doi:10.1186/s13020-025-01281-0)
Supplement: Supplementary file 1 — Additional file 1. [file 13020_2025_1281_MOESM1_ESM.docx]

**Supplementary information**

**Tables:**

**Table S1 Composition of FZJD**

| **Identity document** | **Herbal component (local name)** | **Plant source (scientific name)** | **Dosage (g)** |
| --- | --- | --- | --- |
| a | Codonopsis Radix | *Codonopsis pilosula* (Franch.) Nannf. [Campanulaceae] | 20 |
| b | Bupleuri Radix | *Bupleurum chinense* DC. [Apiaceae] | 15 |
| c | Scutellariae Radix | *Scutellaria baicalensis* Georgi [Lamiaceae] | 15 |
| d | Ephedrae Herba | *Ephedra intermedia* Schrenk & C.A.Mey. [Ephedraceae] | 9 |
| e | Armeniacae Semen Amarum | *Prunus armeniaca* L. [Rosaceae] | 9 |
| f | Gypsum Fibrosum | Not applicable, consists of CaSO_4_·2H_2_O | 30 |
| g | Polygoni Cuspidati Rhizoma Et Radix | *Polygonum cuspidatum* Siebold & Zucc. [Polygonaceae] | 15 |
| h | Coicis Semen | *Coix lacryma-jobi* L. [Poaceae] | 20 |
| i | Amomi Fructus Rotundus | *Amomum kravanh* Pierre ex Gagnep. [Zingiberaceae] | 10 |
| j | Pinelliae Rhizoma Praeparatum Cum Alumine | *Pinellia ternata* (Thunb.) Makino [Araceae] | 10 |
| k | Glycyrrhizae Radix Et Rhizoma Praeparata Cum Melle | *Glycyrrhiza uralensis* Fisch. [Leguminosae] | 6 |

**Note:** The total human equivalent dosage of FZJD is 160 g/day (crud drug equivalent), which was adjusted to 20 g/kg for murine administration based on standard human-to-mouse dose conversation protocols. Except for gypsum fibrosum, the remaining herbal drugs were soaked and decocted twice with 8-fold volumes of water. The resulting decoctions were combined, and gypsum fibrosum was then added to the mixture. The FZJD extract was prepared through filtration, vacuum concentration and freeze-drying, and was stored in aliquots at -20°C. FZJD samples for UPLC/Q-TOF-MS analysis were prepared by sonication in 50% methanol and centrifugation.

**Table S2 Identification of major constituents in FZJD**

| **No.** | **Compound** | **t_R_/min** | **Molecular formula** | **Theoretical**  **(m/z)** | **Observed**  **(m/z)** | **Origin** | **Detectable in plasma** |
| --- | --- | --- | --- | --- | --- | --- | --- |
| 1 | Adenine | 0.757 | C_5_H_5_N_5_ | 136.0623[M+H]^+^ | 136.0468 | j | × |
| 2 | Arginine | 0.793 | C_6_H_14_N_4_O_2_ | 175.1195[M+H]^+^ | 175.1016 | e | × |
| 3 | Valine | 0.841 | C_5_H_11_NO_2_ | 118.0866[M+H]^+^ | 118.0868 | e | × |
| 4 | Sucrose | 0.884 | C_12_H_22_O_11_ | 341.1084[M-H]^-^ | 341.1112 | e | × |
| 5 | D-Gluconic acid | 0.927 | C_6_H_12_O_7_ | 195.0505[M-H]^-^ | 195.0523 | e | × |
| 6 | L-Gluconic acid | 1.105 | C_6_H_12_O_7_ | 195.0505[M-H]^-^ | 195.0523 | e | × |
| 7 | Adenosine | 1.167 | C_10_H_13_N_5_O_4_ | 268.1046[M+H]^+^ | 268.0862 | e | × |
| 8 | Norephedrine | 1.314 | C_9_H_13_NO | 152.1075[M+H]^+^ | 152.0907 | d | × |
| 9 | Norpseudoephedrine | 1.386 | C_9_H_13_NO | 152.1075[M+H]^+^ | 152.0402 | d | × |
| 10 | Guanine | 1.386 | C_5_H_5_N_5_O | 152.0572[M+H]^+^ | 152.0402 | e | × |
| 11 | Guanosine | 1.389 | C_10_H_13_N_5_O_5_ | 284.0995[M+H]^+^ | 284.0792 | e, j | × |
| 12 | Guanine | 1.393 | C_5_H_5_N_5_O | 152.0572[M+H]^+^ | 152.0402 | j | × |
| 13 | Ephedrine | 1.549 | C_10_H_15_NO | 166.1232[M+H]^+^ | 166.1062 | d | √ |
| 14 | Citric acid | 1.569 | C_6_H_8_O_7_ | 191.0192[M-H]^-^ | 191.0207 | e | × |
| 15 | Pseudo-ephedrine | 1.621 | C_10_H_15_NO | 166.1232[M+H]^+^ | 166.1062 | d | √ |
| 16 | Methylephedrine | 1.778 | C_11_H_17_NO | 180.1388[M+H]^+^ | 180.1206 | d | × |
| 17 | Phenylalanine | 1.792 | C_9_H_11_NO_2_ | 166.0868[M+H]^+^ | 166.0692 | e | × |
| 18 | N-benzylidenemethylamine | 1.803 | C_8_H_9_N | 120.0813[M+H]^+^ | 120.0657 | j | × |
| 19 | (2R)-2-(β-D-glucopyranosyloxy)-2-phenylacetamide | 2.79 | C_14_H_19_NO_7_ | 312.1083[M-H]^-^ | 312.1069 | e | × |
| 20 | (R)-(-)-mandelamide | 3.021 | C_8_H_9_NO_2_ | 152.0712[M+H]^+^ | 152.0528 | e | × |
| 21 | O-β-D-gentiobiosyl-D-(-)-mandelamide | 3.061 | C_20_H_29_NO_12_ | 474.1612[M-H]^-^ | 474.1600 | e | × |
| 22 | Tryptophan | 3.22 | C_11_H_12_N_2_O_2_ | 205.0977[M+H]^+^ | 205.0791 | a, e | × |
| 23 | Protocatechuic acid | 3.926 | C_7_H_6_O_4_ | 153.0188[M-H]^-^ | 153.0177 | a | × |
| 24 | Mandelic acid-β-gentiobioside | 4.804 | C_20_H_28_O_13_ | 475.1452[M-H]^-^ | 475.1460 | e | × |
| 25 | Mandelic acid-β-D-glucopyranoside | 4.897 | C_14_H_18_O_8_ | 313.0923[M-H]^-^ | 313.0917 | e | × |
| 26 | Benzyl-β-gentiobioside | 5.268 | C_19_H_28_O_11_ | 431.1553[M-H]^-^ | 431.1563 | e | × |
| 27 | Neochlorogenic acid | 5.368 | C_16_H_18_O_9_ | 353.0873[M-H]^-^ | 353.0867 | b | × |
| 28 | Amygdalin | 5.389 | C_20_H_27_NO_11_ | 456.1506[M-H]^-^ | 456.1541 | e | √ |
| 29 | Catechins | 5.75 | C_15_H_14_O_6_ | 289.0712[M-H]^-^ | 289.0758 | h | × |
| 30 | Tridecanoyl glycine | 5.941 | C_15_H_29_NO_3_ | 272.2226[M+H]^+^ | 272.2040 | j | × |
| 31 | Licochalcone B | 6.018 | C_16_H_14_O_5_ | 285.0763[M-H]^-^ | 285.0649 | k | × |
| 32 | Chlorogenic acid | 6.196 | C_16_H_18_O_9_ | 353.0873[M-H]^-^ | 353.0906 | b | × |
| 33 | Prunasin | 6.332 | C_14_H_17_NO_6_ | 294.0978[M-H]^-^ | 294.1008 | e | √ |
| 34 | Tangshenoside I | 6.617 | C_29_H_42_O_18_ | 677.2293[M-H]^-^ | 677.2303 | a | √ |
| 35 | Schaftoside | 6.696 | C_26_H_28_O_14_ | 565.1557[M+H]^+^ | 565.1364 | k | × |
| 36 | Apiin | 6.704 | C_26_H_28_O_14_ | 565.1557[M+H]^+^ | 565.1364 | j | × |
| 37 | Resveratroloside | 6.91 | C_20_H_22_O_8_ | 389.1236[M-H]^-^ | 389.1252 | g | × |
| 38 | Hexyl β-sophoroside | 7.11 | C_18_H_34_O_11_ | 425.2023[M-H]^-^ | 425.2027 | a | × |
| 39 | Secoisolariciresinol monoglucoside | 7.11 | C_26_H_36_O_11_ | 523.2179[M-H]^-^ | 523.2189 | e | × |
| 40 | 4-O-β-Glucopyranosyl-*cis*-coumaric acid | 7.267 | C_15_H_18_O_8_ | 325.0923[M-H]^-^ | 325.0933 | a | × |
| 41 | Hexyl-β-gentiobioside | 7.367 | C_18_H_34_O_11_ | 425.2023[M-H]^-^ | 425.2027 | a | × |
| 42 | Chrysin 6-C-arabinoside 8-C-glucoside | 7.69 | C_26_H_28_O_13_ | 549.1608[M+H]^+^ | 549.1357 | c | × |
| 43 | Liquiritin apioside | 7.731 | C_26_H_30_O_13_ | 549.1608[M-H]^-^ | 549.1601 | k | × |
| 44 | Isovitexin-2''-O-rhamnoside | 7.861 | C_27_H_30_O_14_ | 579.1714[M+H]^+^ | 579.1482 | d | × |
| 45 | Isoliquiritin | 7.874 | C_21_H_22_O_9_ | 417.1186[M-H]^-^ | 417.1212 | k | × |
| 46 | Penta-aldosylglucose hexanoside | 8.003 | C_17_H_32_O_10_ | 395.1917[M-H]^-^ | 395.1919 | a | × |
| 47 | p-coumaric acid | 8.024 | C_9_H_8_O_3_ | 163.0395[M-H]^-^ | 163.0409 | a | × |
| 48 | Liguiritigenin-7-O-D-apiosyl-4'-O-D-glucoside | 8.173 | C_26_H_30_O_13_ | 549.1608[M-H]^-^ | 549.1649 | k | × |
| 49 | Liquiritin | 8.295 | C_21_H_22_O_9_ | 417.1186[M-H]^-^ | 417.1212 | k | √ |
| 50 | Chrysin 6-C-glucoside 8-C-arabinoside | 8.333 | C_26_H_28_O_13_ | 549.1608[M+H]^+^ | 549.1404 | c | × |
| 51 | Polydatin | 8.366 | C_20_H_22_O_8_ | 389.1236[M-H]^-^ | 389.1252 | g | √ |
| 52 | Coixol | 8.639 | C_8_H_7_NO_3_ | 166.0504[M-H]^-^ | 166.0349 | h | √ |
| 53 | β-D-glucose hexenol glucoside | 9.194 | C_12_H_22_O_6_ | 261.1338[M-H]^-^ | 261.1343 | a | × |
| 54 | Saikosaponin V | 9.237 | C_53_H_86_O_24_ | 1151.5509[M+HCOO]^-^ | 1151.5452 | b | × |
| 55 | Rutin | 9.426 | C_27_H_30_O_16_ | 611.1612[M+H]^+^ | 611.1426 | d | × |
| 56 | Saikosaponin Q | 9.644 | C_48_H_78_O_19_ | 1003.5149[M+HCOO]^-^ | 1003.5035 | b | × |
| 57 | Saikosaponin V1 | 9.944 | C_47_H_76_O_19_ | 989.4982[M+HCOO]^-^ | 989.4886 | b | × |
| 58 | Narcissoside | 10.38 | C_28_H_32_O_16_ | 623.1612[M-H]^-^ | 623.1603 | b | × |
| 59 | Azelaic acid | 10.644 | C_9_H_16_O_4_ | 187.0970[M-H]^-^ | 187.0984 | a, e | √ |
| 60 | Isochlorogenic acid B | 10.801 | C_25_H_24_O_12_ | 515.1190[M-H]^-^ | 515.1141 | b | × |
| 61 | Lobetyolin | 11.001 | C_20_H_28_O_8_ | 395.1706[M-H]^-^ | 395.1716 | a | √ |
| 62 | Isorhamnetin-3-O-β-D-glucoside | 11.251 | C_22_H_22_O_12_ | 477.1033[M-H]^-^ | 477.1032 | b | × |
| 63 | 6''-Acetylliquiritin | 11.587 | C_23_H_24_O_10_ | 459.1291[M-H]^-^ | 459.1287 | k | × |
| 64 | Isochlorogenic acid A | 12.114 | C_25_H_24_O_12_ | 515.1190[M-H]^-^ | 515.1095 | b | × |
| 65 | 7-Methoxyliquiritin | 12.259 | C_22_H_22_O_9_ | 431.1342[M+H]^+^ | 431.1129 | k | × |
| 66 | Emodin-8-glucoside | 12.907 | C_21_H_20_O_10_ | 431.0978[M-H]^-^ | 431.0968 | g | × |
| 67 | Isochlorogenic acid C | 13.207 | C_25_H_24_O_12_ | 515.1190[M-H]^-^ | 515.1188 | b | × |
| 68 | Isoliquiritin apioside | 13.271 | C_26_H_30_O_13_ | 549.1608[M-H]^-^ | 549.1601 | k | × |
| 69 | 5,7,2',5'-Tetrahydroxy-8,6'-dimethoxyflavone | 13.644 | C_17_H_14_O_8_ | 347.0767[M+H]^+^ | 347.0563 | c | × |
| 70 | Resveratrol | 13.657 | C_14_H_12_O_3_ | 227.0708[M-H]^-^ | 227.0709 | g | √ |
| 71 | 5,7,2'-Trihydroxy-6-methoxyflavone-7-O-glucuronide | 13.988 | C_22_H_20_O_12_ | 477.1033[M+H]^+^ | 477.0835 | c | × |
| 72 | Neoliquiritin | 14.036 | C_21_H_22_O_9_ | 417.1186[M-H]^-^ | 417.1212 | k | × |
| 73 | Baicalin | 14.273 | C_21_H_18_O_11_ | 447.0927[M+H]^+^ | 447.0717 | c | × |
| 74 | Neoisoliquiritin | 14.713 | C_21_H_22_O_9_ | 417.1186[M-H]^-^ | 417.117 | k | × |
| 75 | Liquiritigenin | 15.163 | C_15_H_12_O_4_ | 255.0657[M-H]^-^ | 255.0677 | k | √ |
| 76 | Licorice glycoside A | 15.734 | C_36_H_38_O_16_ | 725.2082[M-H]^-^ | 725.2039 | k | × |
| 77 | Licorice glycoside B | 15.856 | C_35_H_36_O_15_ | 695.1976[M-H]^-^ | 695.1954 | k | × |
| 78 | Aloe-emodin-8-O-β-D-glucopyranoside | 16.163 | C_20_H_24_O_9_ | 407.1342[M-H]^-^ | 407.1355 | g | × |
| 79 | 5,7,8-Trihydroxy-6-methoxyflavone-7-O-glucuronide | 17.257 | C_22_H_20_O_12_ | 477.1033[M+H]^+^ | 477.0835 | c | × |
| 80 | Emodin-1-O-glucoside | 17.63 | C_21_H_20_O_10_ | 431.0978[M-H]^-^ | 431.0968 | g | × |
| 81 | Chrysin-7-O-glucuronide | 17.814 | C_21_H_18_O_10_ | 431.0978[M+H]^+^ | 431.0747 | c | × |
| 82 | Oroxylin A-7-O-glucuronide | 17.95 | C_22_H_20_O_11_ | 461.1084[M+H]^+^ | 461.0851 | c | × |
| 83 | 5,6,7-Trihydroxy-8-methoxyflavone-7-O-glucuronide | 18.6 | C_22_H_20_O_12_ | 477.1033[M+H]^+^ | 477.0790 | c | × |
| 84 | Oroxindin | 19.543 | C_22_H_20_O_11_ | 461.1084[M+H]^+^ | 461.0851 | c | √ |
| 85 | 5,7-Dihydroxy-6,8-dimethoxyflavone-7-O-glucuronide | 20.199 | C_23_H_22_O_12_ | 491.1190[M+H]^+^ | 491.0978 | c | × |
| 86 | Hydroxysaikosaponin C | 20.339 | C_48_H_80_O_18_ | 989.5354[M+HCOO]^-^ | 989.5273 | b | × |
| 87 | Hydroxysaikosaponin A | 21.575 | C_42_H_70_O_14_ | 843.4763[M+HCOO]^-^ | 843.4705 | b | × |
| 88 | Aloe-emodin-8-O-(6'-acetyl)-glucoside | 22.174 | C_22_H_26_O_12_ | 449.1448[M-H]^-^ | 449.1483 | g | × |
| 89 | Physcion 8-glucoside | 22.246 | C_22_H_22_O_10_ | 445.1135[M-H]^-^ | 445.1125 | g | × |
| 90 | Bupleuroside X | 22.41 | C_48_H_78_O_18_ | 987.5192[M+HCOO]^-^ | 987.5120 | b | × |
| 91 | 9,12,13-Trihydroxy-10,15-octadecadienoic acid | 22.639 | C_18_H_32_O_5_ | 327.2171[M-H]^-^ | 327.2162 | a | × |
| 92 | Norwogonin | 23.056 | C_15_H_10_O_5_ | 271.0606[M+H]^+^ | 271.0604 | c | × |
| 93 | Apigenin | 23.143 | C_15_H_10_O_5_ | 269.0450[M-H]^-^ | 269.0491 | j | √ |
| 94 | Bupleuroside I | 23.181 | C_48_H_78_O_18_ | 987.5192[M+HCOO]^-^ | 987.5120 | b | × |
| 95 | Licoricesaponin A3 | 23.374 | C_48_H_72_O_21_ | 983.4488[M-H]^-^ | 983.4491 | k | √ |
| 96 | Baicalein | 23.512 | C_15_H_10_O_5_ | 271.0606[M+H]^+^ | 271.0402 | c | √ |
| 97 | 2-Amino-2-tridecylpropane-1,3-diol | 23.74 | C_16_H_35_NO_2_ | 274.2746[M-H]^-^ | 274.2543 | j | × |
| 98 | 9,12,13-Trihydroxy-10-octadecenoic acid | 23.859 | C_18_H_34_O_5_ | 329.2328[M-H]^-^ | 329.2344 | a | × |
| 99 | Pinellia acid | 23.859 | C_18_H_34_O_5_ | 329.2328[M-H]^-^ | 329.2344 | b | × |
| 100 | Phytosphingosine | 23.919 | C_18_H_39_NO_3_ | 318.3008[M-H]^-^ | 318.2798 | j | × |
| 101 | Funtumine | 23.926 | C_21_H_35_NO | 318.2797[M+H]^+^ | 318.2798 | j | × |
| 102 | Saikosaponin C | 24.131 | C_48_H_78_O_17_ | 971.5222[M+HCOO]^-^ | 971.5213 | b | √ |
| 103 | Physcion-8-O-(6'-acetyl)-glucoside | 24.237 | C_24_H_24_O_11_ | 487.1240[M-H]^-^ | 487.1256 | g | × |
| 104 | Saikosaponin F | 24.449 | C_48_H_80_O_17_ | 973.5381[M+HCOO]^-^ | 973.5394 | b | × |
| 105 | 6-Hydroxywogonin | 24.49 | C_16_H_12_O_6_ | 301.0712[M+H]^+^ | 301.0502 | c | × |
| 106 | Isoliquiritigenin | 24.62 | C_15_H_12_O_4_ | 255.0657[M-H]^-^ | 255.0677 | k | × |
| 107 | Licoricesaponin E2 | 24.635 | C_42_H_60_O_16_ | 819.3803[M-H]^-^ | 819.3795 | k | × |
| 108 | Licoricesaponin G2 | 24.936 | C_42_H_62_O_17_ | 839.4065[M+H]^+^ | 839.3847 | k | √ |
| 109 | Uralsaponin U | 25.186 | C_42_H_62_O_17_ | 839.4065[M+H]^+^ | 839.3906 | k | × |
| 110 | Saikosaponin A | 25.264 | C_42_H_68_O_13_ | 825.4650[M+HCOO]^-^ | 825.4636 | b | √ |
| 111 | Saikosaponin B2 | 25.289 | C_42_H_68_O_13_ | 825.4650[M+HCOO]^-^ | 825.4636 | b | √ |
| 112 | Licoricesaponin B2 | 25.35 | C_42_H_64_O_15_ | 807.4167[M-H]^-^ | 807.4183 | k | × |
| 113 | Saikosaponin D | 25.357 | C_42_H_68_O_13_ | 825.4650[M+HCOO]^-^ | 825.4636 | b | √ |
| 114 | Glycyrrhizic acid | 25.45 | C_42_H_62_O_16_ | 823.4116[M+H]^+^ | 823.3970 | k | √ |
| 115 | Wogonin | 25.583 | C_16_H_12_O_5_ | 285.0763[M+H]^+^ | 285.0564 | c | √ |
| 116 | 2"-O-Acetylsaikosaponin A | 25.665 | C_44_H_70_O_14_ | 867.4744[M+HCOO]^-^ | 867.4762 | b | × |
| 117 | Uralsaponin B | 25.721 | C_42_H_62_O_16_ | 823.4116[M+H]^+^ | 823.3970 | k | × |
| 118 | Skullcapflavone II | 25.74 | C_19_H_18_O_8_ | 375.1080[M+H]^+^ | 375.0854 | c | × |
| 119 | 1,8-cineole | 25.799 | C_10_H_18_O | 155.1436[M+H]^+^ | 155.1392 | i | √ |
| 120 | 3"-O-Acetylsaikosaponin A | 25.828 | C_44_H_70_O_14_ | 867.4744[M+HCOO]^-^ | 867.4762 | b | × |
| 121 | Chrysin | 25.861 | C_15_H_10_O_4_ | 255.0657[M+H]^+^ | 255.0479 | c | × |
| 122 | Prosaikogenin F | 25.971 | C_36_H_58_O_8_ | 663.4125[M+HCOO]^-^ | 663.4136 | b | × |
| 123 | Oroxylin A | 26.004 | C_6_H_12_O_5_ | 285.0763[M+H]^+^ | 285.0564 | c | √ |
| 124 | Saikosaponin E | 26.121 | C_42_H_68_O_12_ | 809.4701[M+HCOO]^-^ | 809.4678 | b | × |
| 125 | 4''-O-acetylsaikosaponin A | 26.278 | C_44_H_70_O_14_ | 867.4744[M+HCOO]^-^ | 867.4762 | b | × |
| 126 | 6''-O-acetylsaikosaponin A | 26.328 | C_44_H_70_O_14_ | 867.4744[M+HCOO]^-^ | 867.4762 | b | × |
| 127 | Glycycoumarin | 26.371 | C_21_H_20_O_6_ | 367.1182[M-H]^-^ | 367.1186 | k | × |
| 128 | Gancaonin N | 26.378 | C_21_H_20_O_6_ | 367.1182[M-H]^-^ | 367.1186 | k | √ |
| 129 | Licoricesaponin J2 | 26.492 | C_42_H_64_O_16_ | 825.4273[M+H]^+^ | 825.4128 | k | × |
| 130 | Licoflavonol | 26.564 | C_20_H_18_O_6_ | 353.1025[M-H]^-^ | 353.1022 | k | × |
| 131 | Licoisoflavone B | 26.657 | C_20_H_16_O_6_ | 351.0869[M-H]^-^ | 351.0886 | k | × |
| 132 | Saikosaponin G | 26.664 | C_42_H_68_O_13_ | 825.4650[M+HCOO]^-^ | 825.4636 | b | × |
| 133 | Isolicoflavonol | 27.064 | C_20_H_18_O_6_ | 353.1025[M-H]^-^ | 353.1021 | k | × |
| 134 | Gancaonin L | 27.278 | C_20_H_18_O_6_ | 353.1025[M-H]^-^ | 353.1021 | k | × |
| 135 | 9,10-Dihydroxy-12-octadecenoic acid | 27.342 | C_18_H_34_O_4_ | 313.2379[M-H]^-^ | 313.2367 | a | × |
| 136 | LPC (18:3) | 27.343 | C_26_H_48_NO_7_P | 518.3247[M+H]^+^ | 518.3081 | k | × |
| 137 | Emodin | 27.849 | C_15_H_10_O_5_ | 269.0450[M-H]^-^ | 269.0461 | g | √ |
| 138 | LPE (18:2) | 27.996 | C_23_H_44_NO_7_P | 478.2934[M+H]^+^ | 478.2734 | k | × |
| 139 | Semilicoisoflavone B | 28.031 | C_20_H_16_O_6_ | 351.0869[M-H]^-^ | 351.0848 | k | × |
| 140 | LPC (18:2) | 28.039 | C_26_H_50_NO_7_P | 520.3403[M+H]^+^ | 520.3184 | j | × |
| 141 | Glyceryl linolenate | 28.189 | C_21_H_36_O_4_ | 353.2692[M+H]^+^ | 353.2495 | j | × |
| 142 | LPC (16:1) | 28.546 | C_24_H_50_NO_7_P | 496.3403[M+H]^+^ | 496.3188 | j | × |
| 143 | 9,10-Epoxy-12-octadecenoic acid | 28.848 | C_18_H_32_O_3_ | 295.2273[M-H]^-^ | 295.2292 | a | × |
| 144 | LPC (18:1) | 28.874 | C_26_H_52_NO_7_P | 522.3560[M+H]^+^ | 522.3373 | j | × |
| 145 | 1-Linoleoyl glycerol | 28.924 | C_21_H_38_O_4_ | 355.2848[M+H]^+^ | 331.2633 | j | × |
| 146 | Aloe-emodin | 28.97 | C_15_H_10_O_5_ | 269.0450[M-H]^-^ | 269.0461 | g | √ |
| 147 | 2,4-decadienal | 29.449 | C_10_H_16_O | 152.1201[M+H]+ | 152.1122 | b | √ |
| 148 | Glycyrrhetic acid | 29.713 | C_30_H_46_O_4_ | 469.3318[M-H]^-^ | 469.3414 | k | √ |
| 149 | Cholesterol | 29.913 | C_27_H_46_O | 385.3470[M-H]^-^ | 385.3369 | h | √ |

**Figures:**

**
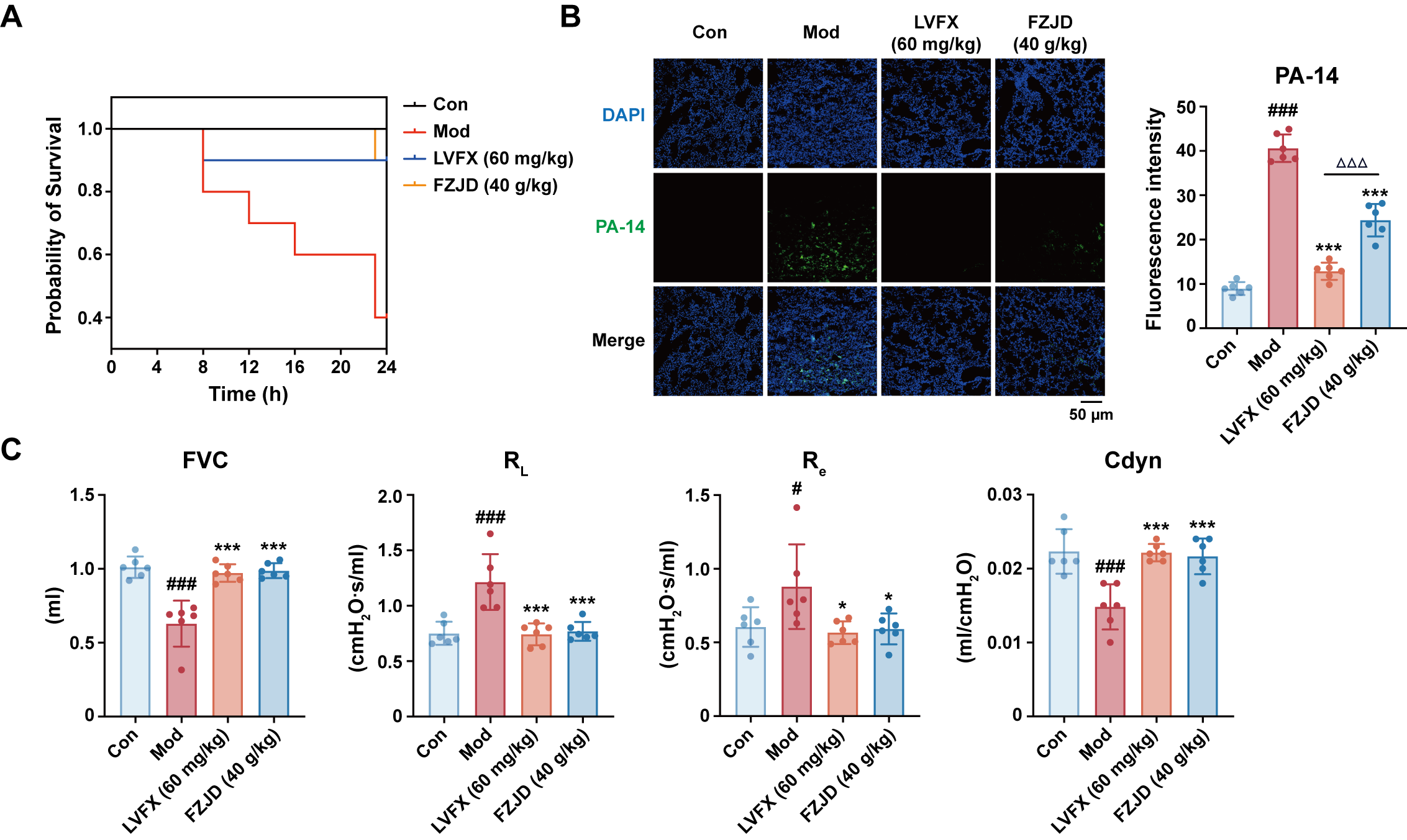
**

**Fig. S1** Effect of FZJD on survival, pulmonary bacterial burden and lung function in PA-14-induced pneumonia. (**A**) Survival curves over 24 h following intranasal instillation of Pseudomonas aeruginosa PA-14 (20 μl, 5 × 10^7^ CFU/ml). Con, uninfected control; Mod, infected + vehicle; LVFX, levofloxacin 60 mg/kg (i.p.); FZJD, 40 g/kg (p.o.); *n* = 10. (**B**) Confocal images of lung sections stained with PA-14 (green) and DAPI (blue) at 24 h post-infection (20 μl, 1 × 10^7^ CFU/ml; ###*p* < 0.001, vs. Con group; ****p* < 0.001, vs. Mod group; △△△p < 0.001, vs. LVFX group; *n* = 6). (**C**) Pulmonary function parameters measured at 24 h post-infection (20 μl, 1 × 10^7^ CFU/ml) including forced vital capacity (FVC), airway resistance (RL), respiratory system elastance (Re), and dynamic compliance (Cdyn) (#*p* < 0.05, ###*p* < 0.001, vs. Con group; **p* < 0.001, ****p* < 0.001, vs. Mod group; *n* = 6).

**
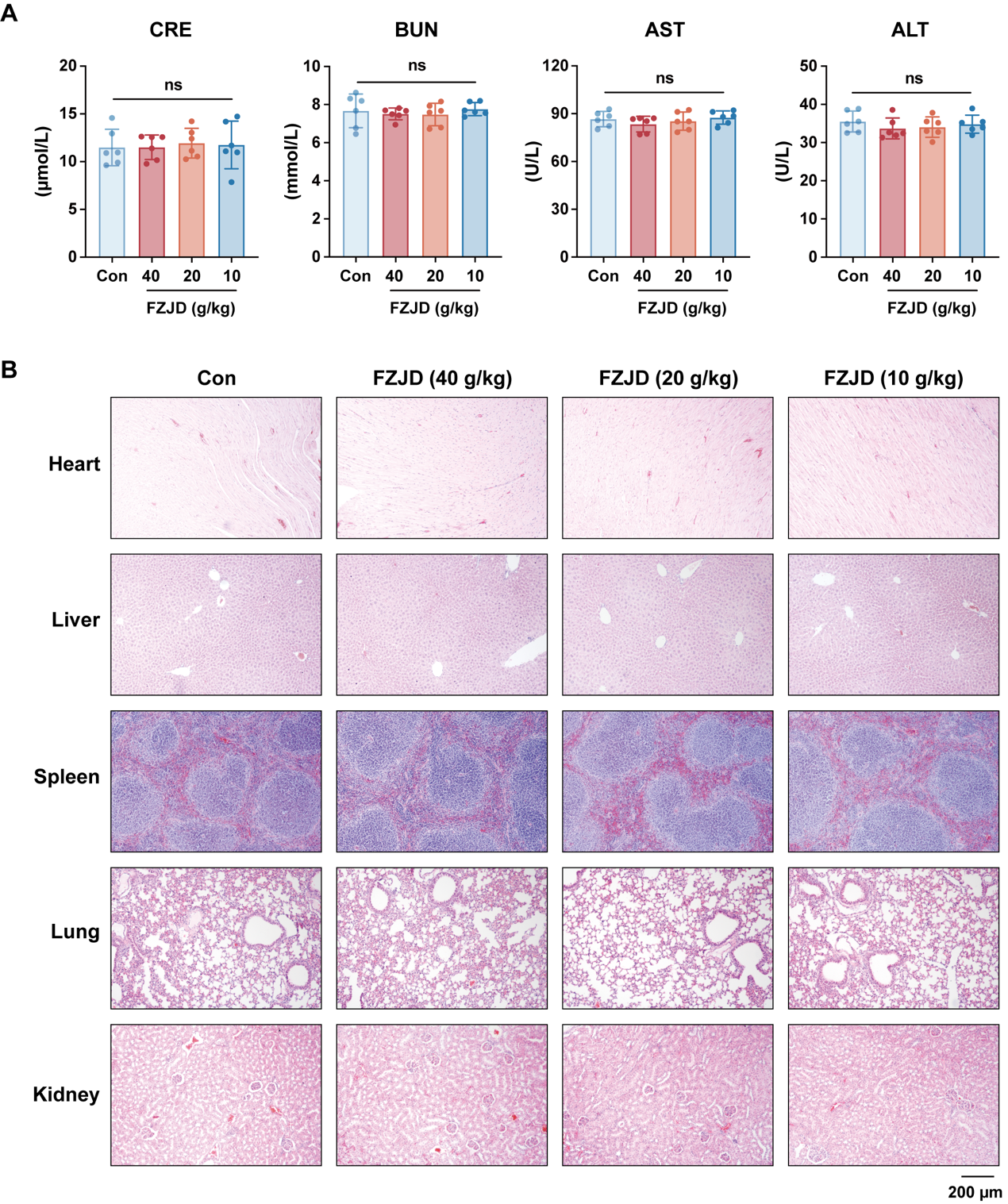
**

**Fig. S2** Safety evaluation of orally administered FZJD in mice. (**A**) Serum biochemical indices including creatinine (CRE), blood urea nitrogen (BUN), aspartate aminotransferase (AST), and alanine aminotransferase (ALT) in control and FZJD-treated groups (10, 20, and 40 g/kg; *n* = 6). No significant differences were observed (*p* > 0.05). (**B**) Representative H&E micrographs of heart, liver, spleen, lung, and kidney from the same groups.

**
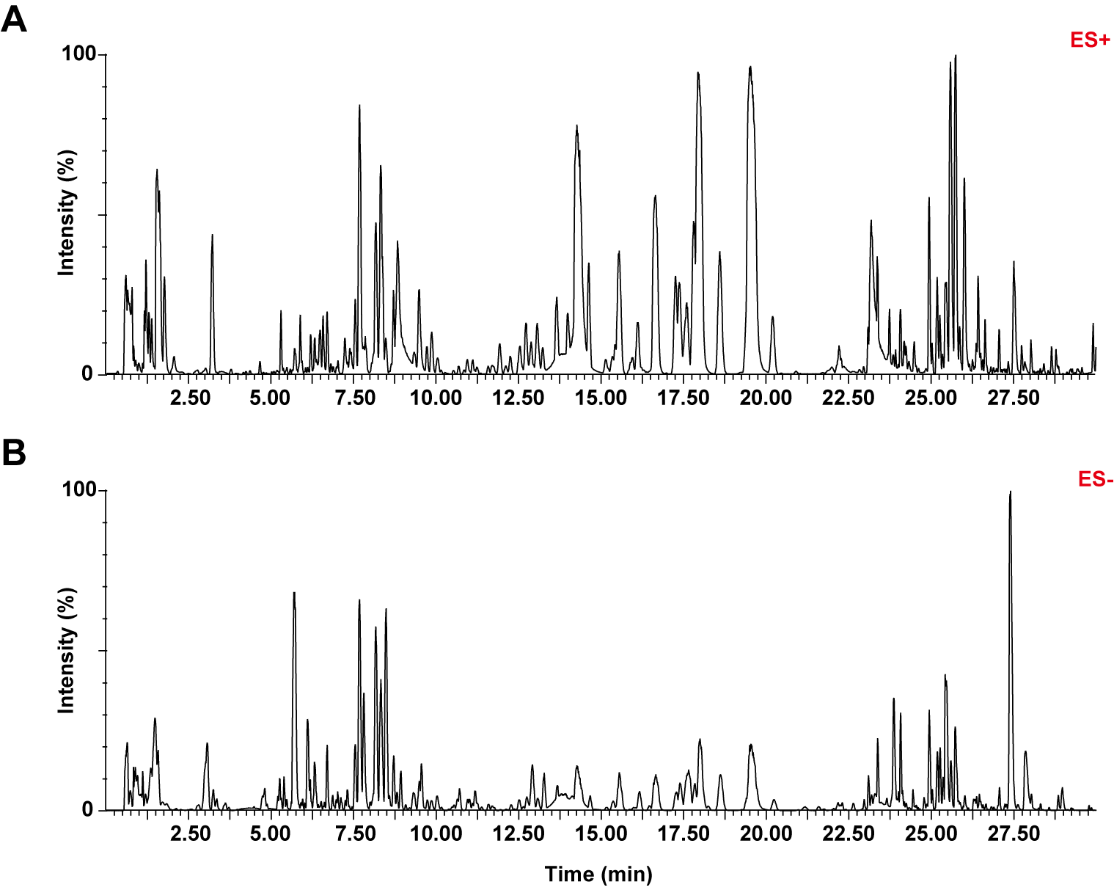
**

**Fig. S3** Ion chromatograms of FZJD in (**A**) positive and (**B**) negative ion modes.

**
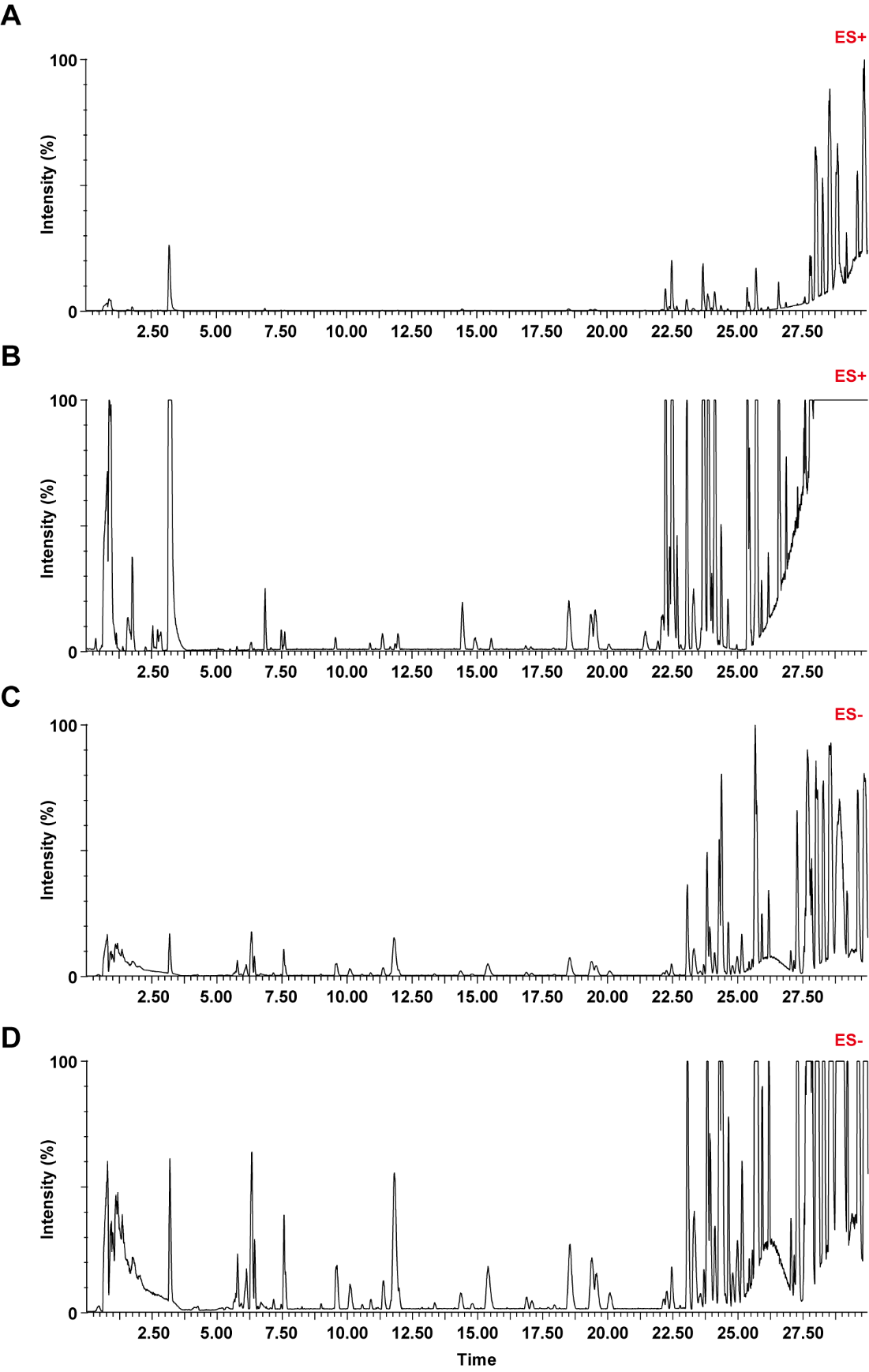
**

**Fig. S4** Ion chromatograms of plasma sample from FZJD-administrated rats. (**A**) Original positive ion mode chromatogram and (**B**) magnified view of peaks; (**C**) Original negative ion mode chromatogram and (**D**) magnified view of peaks.


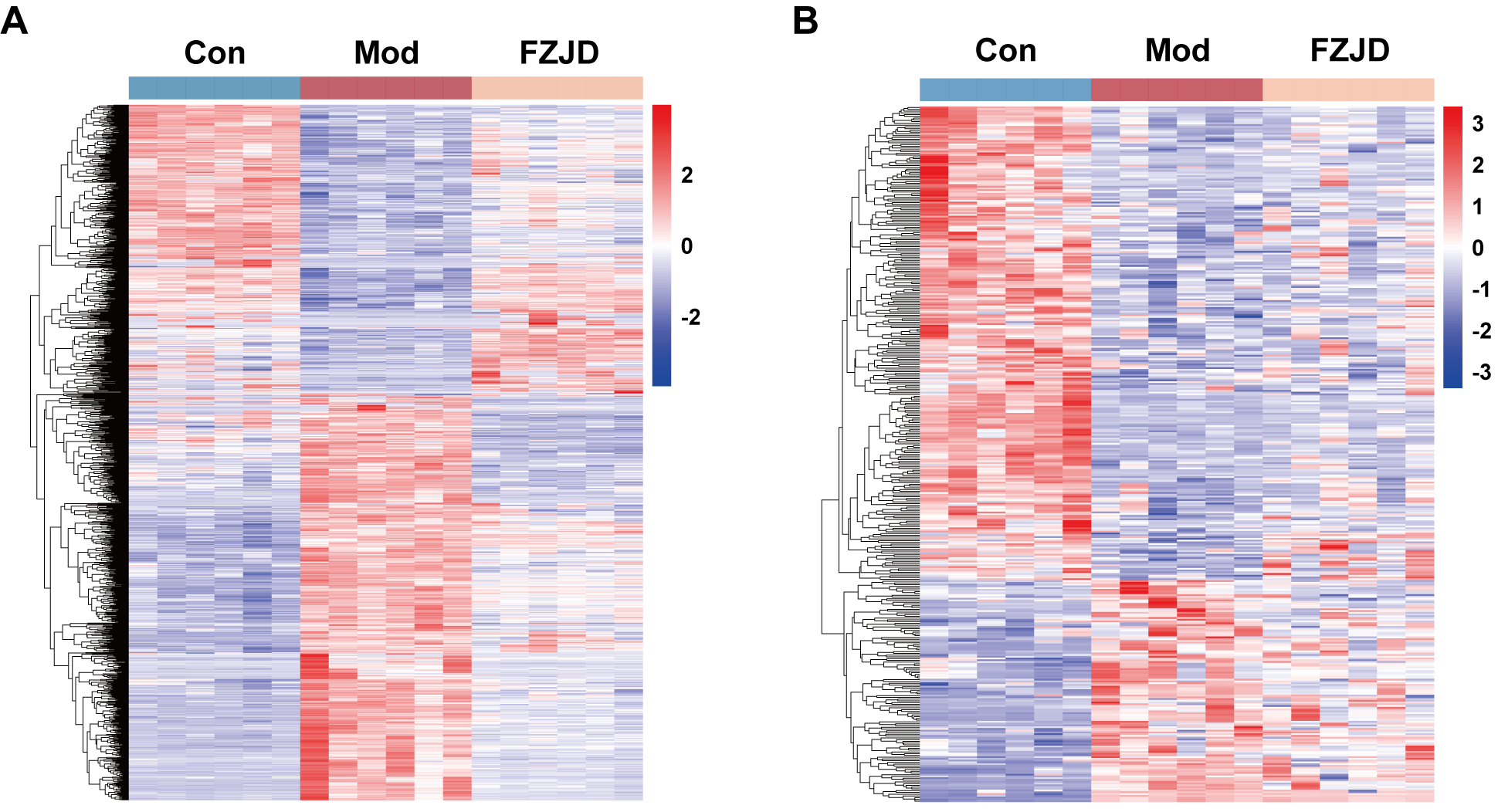


**Fig. S5** Normalized heatmaps of (**A**) 1062 reversed DEPs in lung tissues used for proteomic analysis and (**B**) 395 reversed DEMs in plasma used for metabolomic analysis.


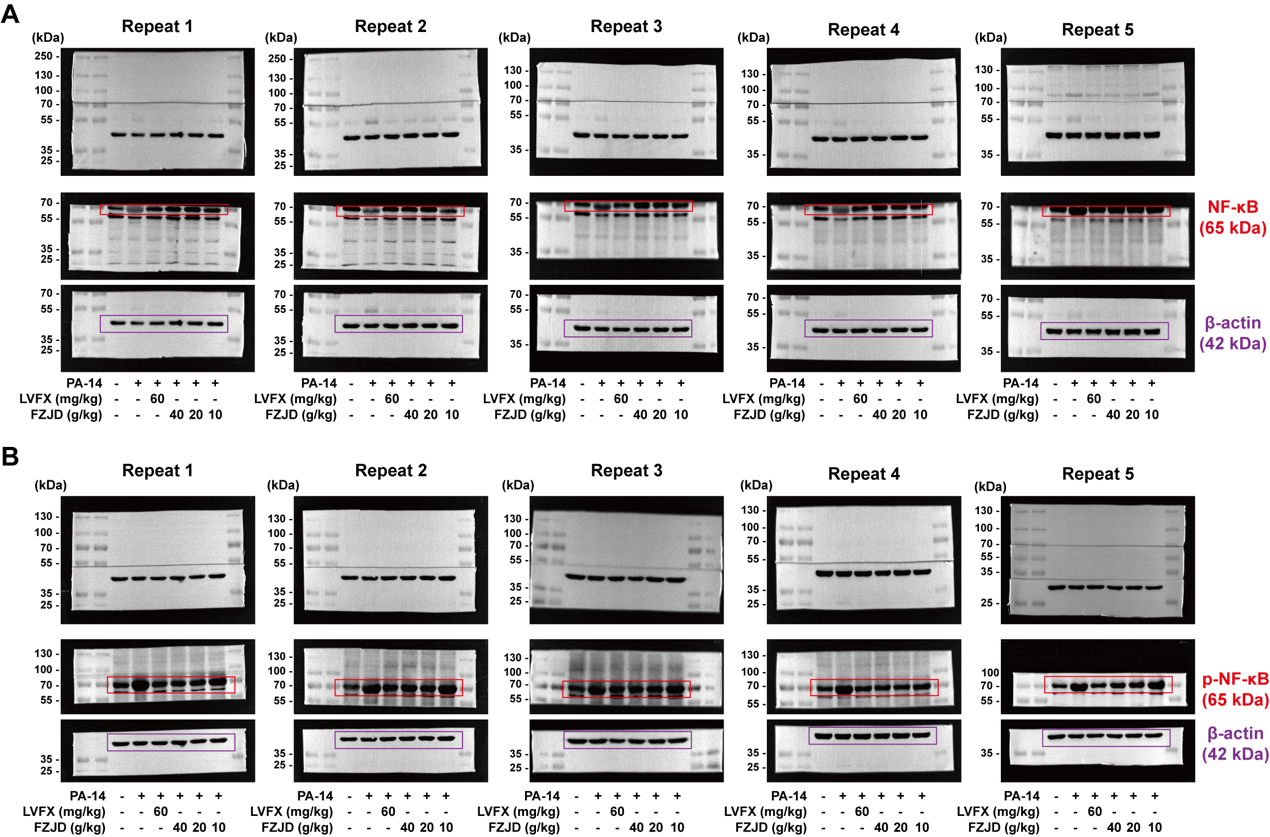


**Fig. S6** Original Western blots of (**A**) NF-κB and (**B**) p-NF-κB (Ser536) in Fig. 4B, and β-actin was selected as loading control (*n* = 5).


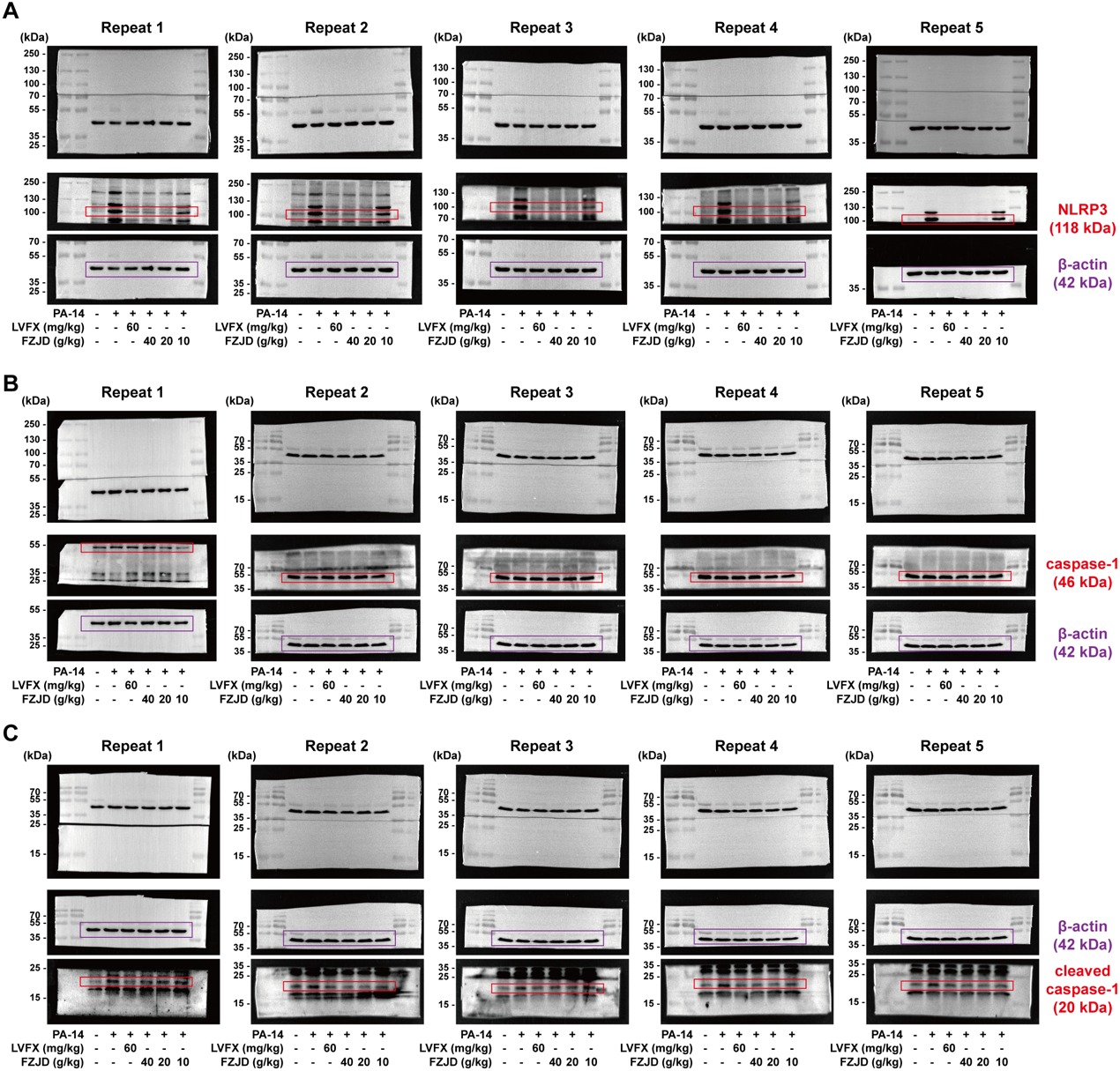


**Fig. S7** Original Western blots of (**A**) NLRP3, (**B**) caspase-1 and (**C**) cleaved caspase-1 in Fig. 4B, and β-actin was selected as loading control (*n* = 5).


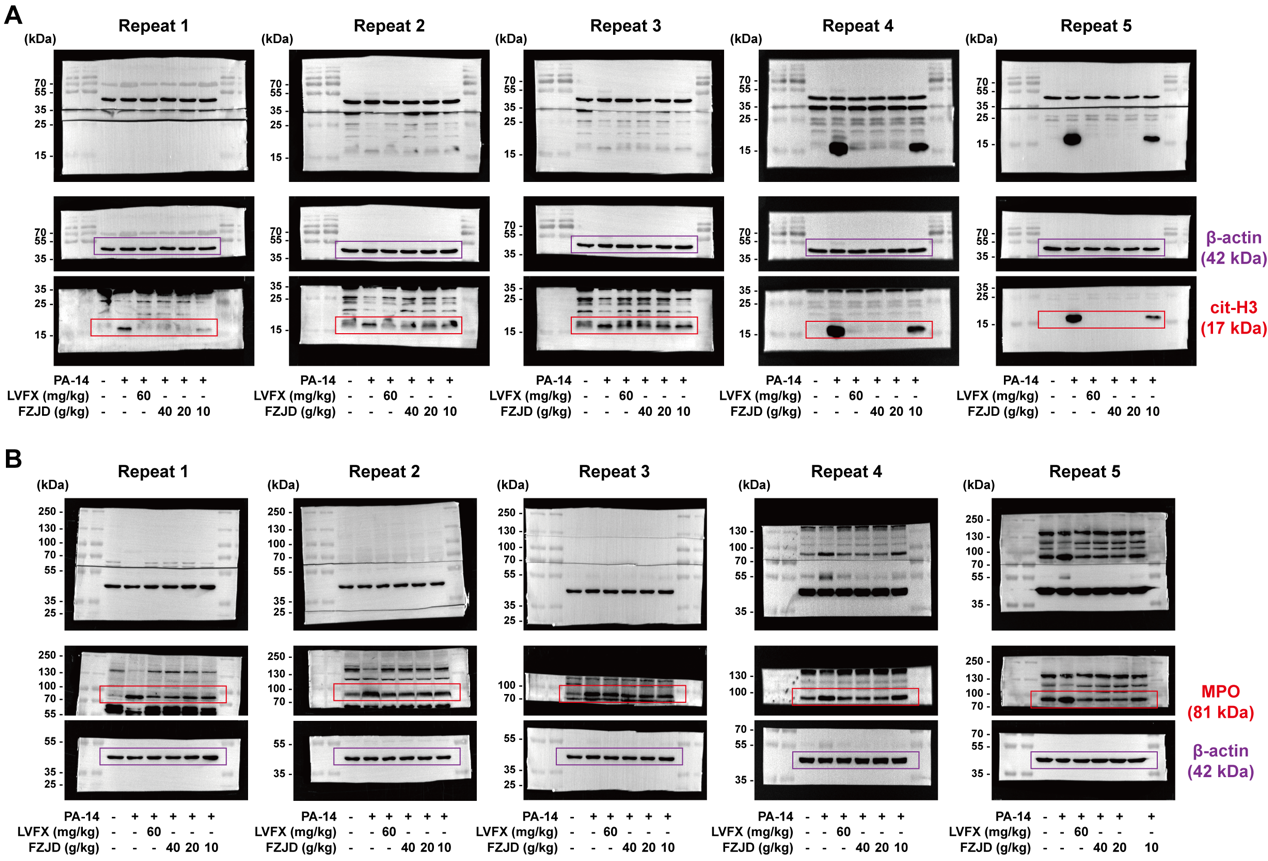


**Fig. S8** Original Western blots of (**A**) cit-H3 and (**B**) MPO in Fig. 4E, and β-actin was selected as loading control (*n* = 5).

As shown in Fig. S8, cell viability assays of 27 representative plasma exposure components in 293T and RAW 2647.7 cells revealed that saikosaponin A and saikosaponin D exhibited significant viability inhibition (10 μM) in both cell lines, with emodin additionally demonstrating marked cytotoxicity in RAW 264.7 cells at equivalent concentration. Based on these findings, we have standardized a 1 μM administration dose for subsequent bioactivity screening experiments across all 27 components.

**
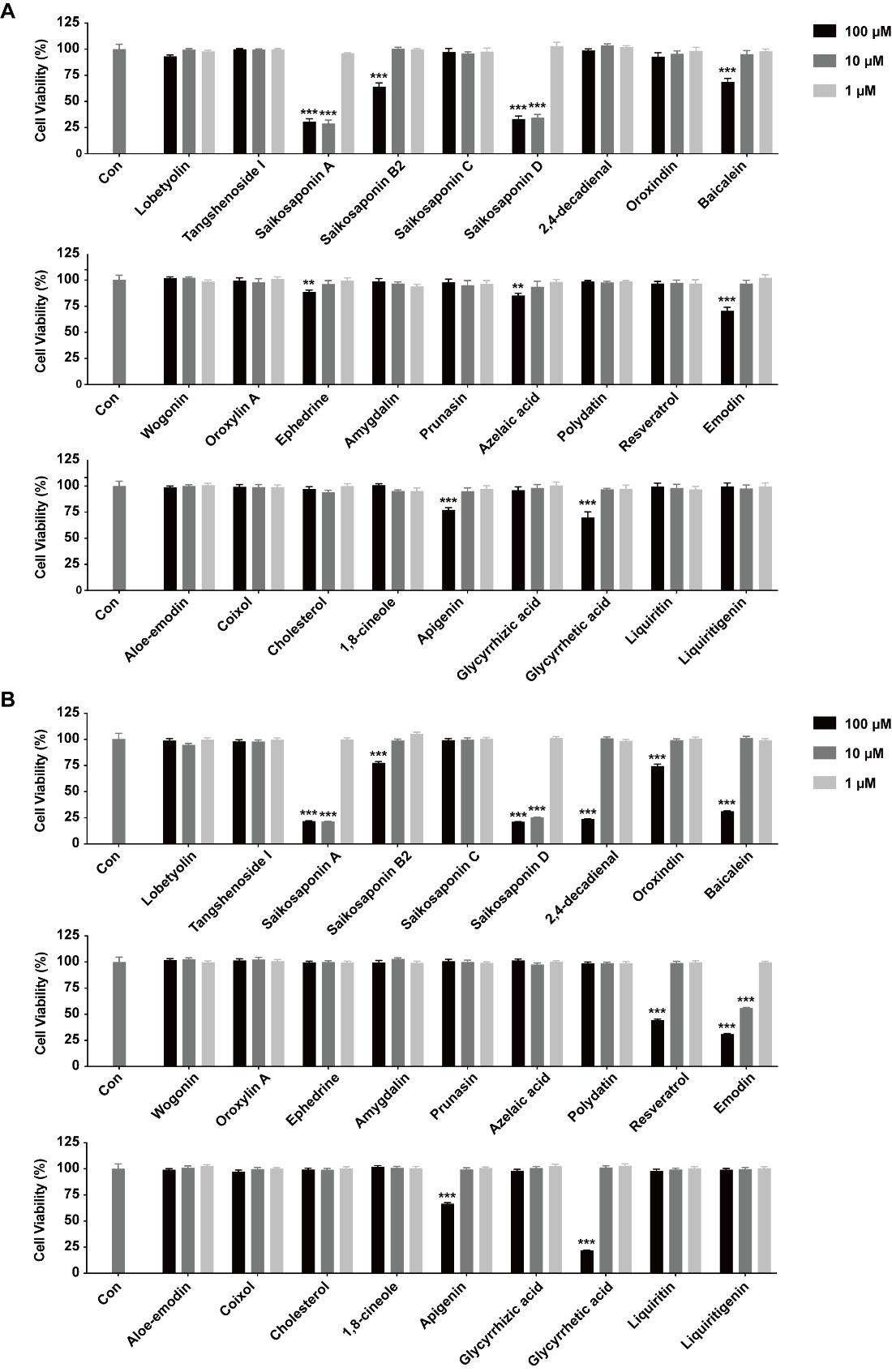
**

**Fig. S9** CCK8 assay evaluating the effects of 27 representative components on cell viability in (**A**) 293T and (**B**) RAW 264.7 cells. (***p* < 0.01, ****p* < 0.001, *vs.* Con group, *n* = 3)


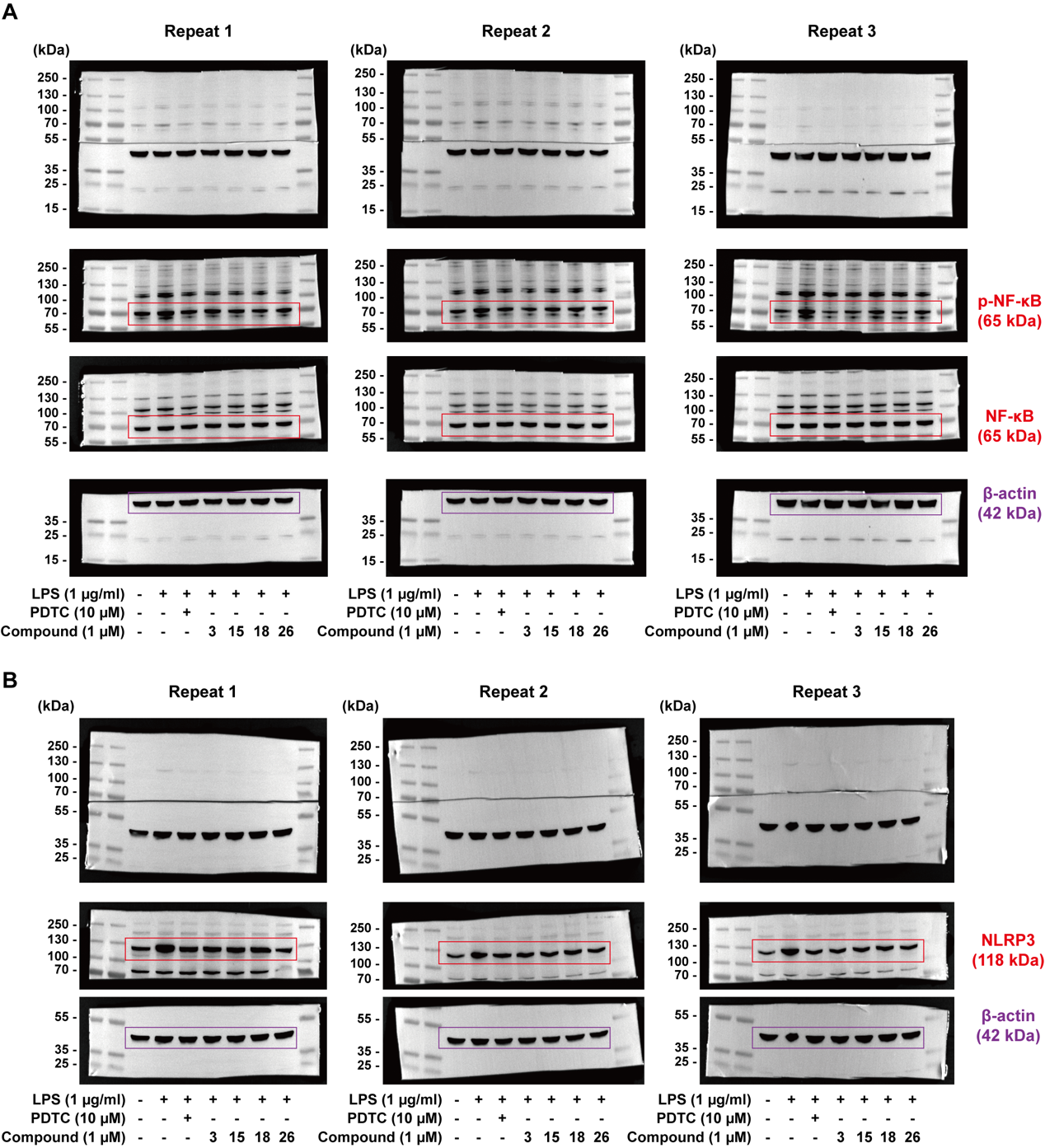


**Fig. S10** Original Western blots of (**A**) p-NF-κB (Ser536) and NF-κB, and (**B**) NLRP3 in Fig. 5B, and β-actin was selected as loading control (*n* = 3).


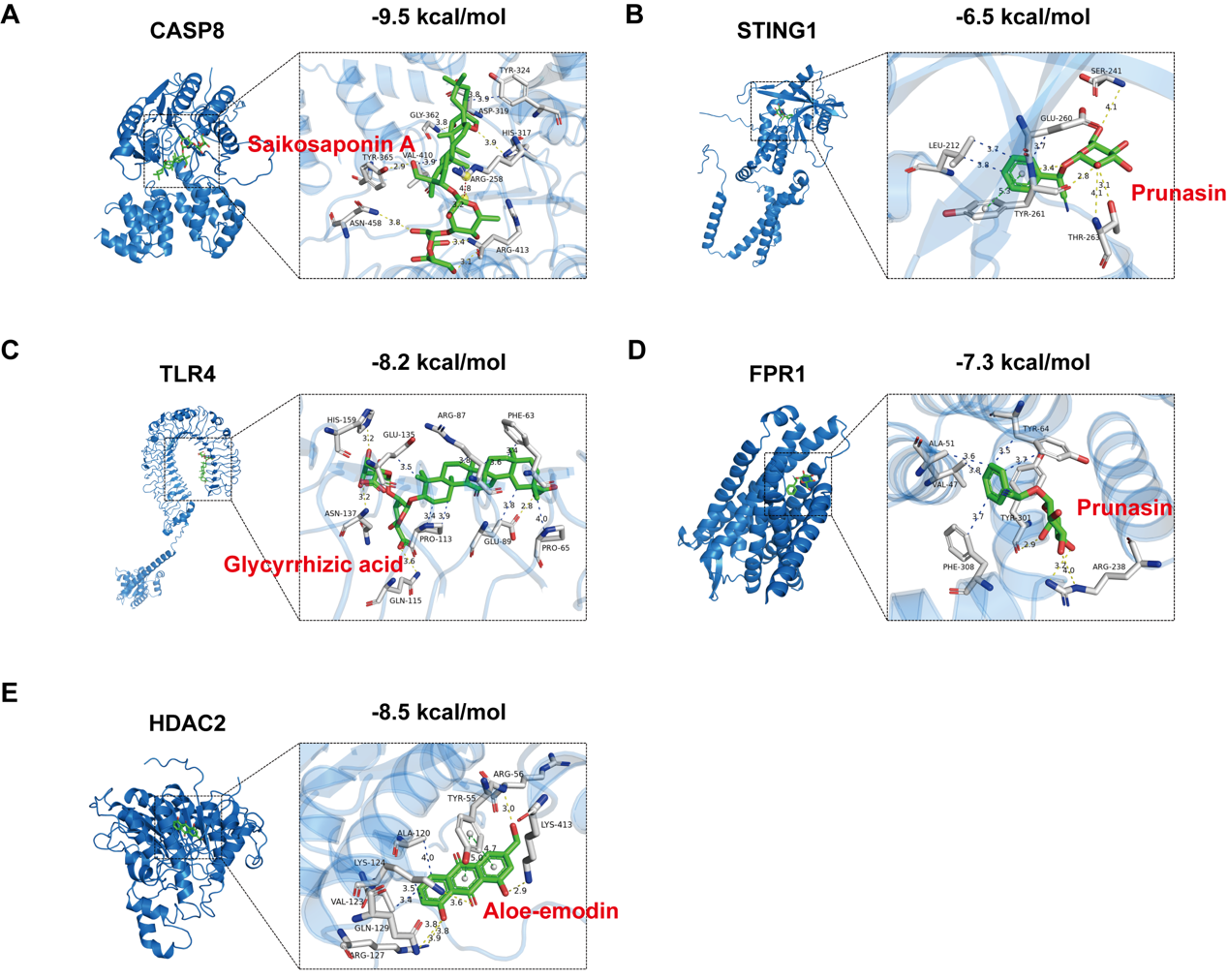


**Fig. S11** Molecular docking based on metwork pharmacology prediction. Dockings of (**A**) saikosaponin A with CASP8, (**B**) prunasin with STING1, (**C**) glycyrrhizic acid with TLR4, (**D**) prunasin with FPR1, and (**E**) aloe-emodin with HDAC2.

**
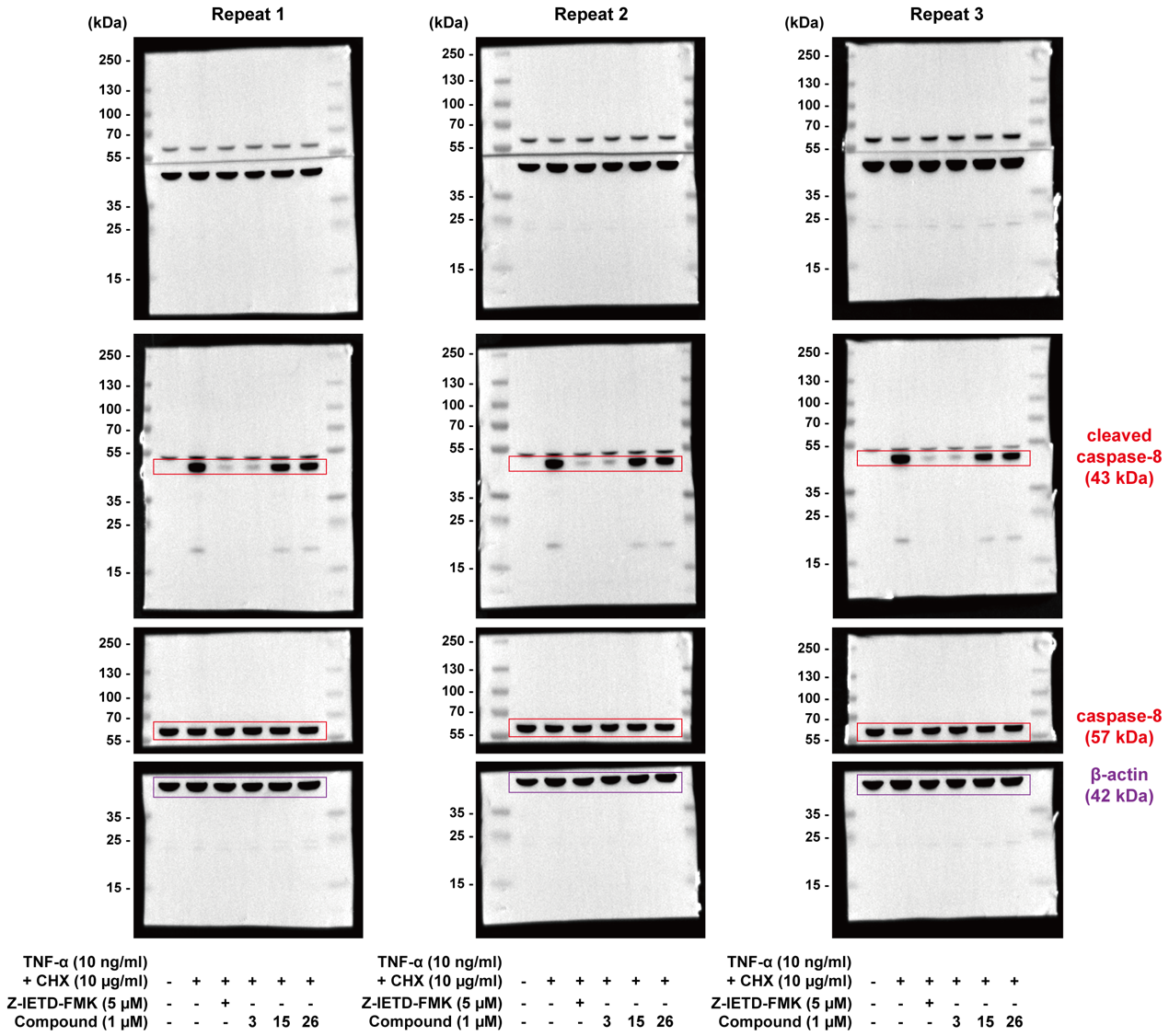
**

**Fig. S12** Original Western blots of cleaved caspase-8 and caspase-8 in Fig. 5D, and β-actin was selected as loading control (*n* = 3).

**
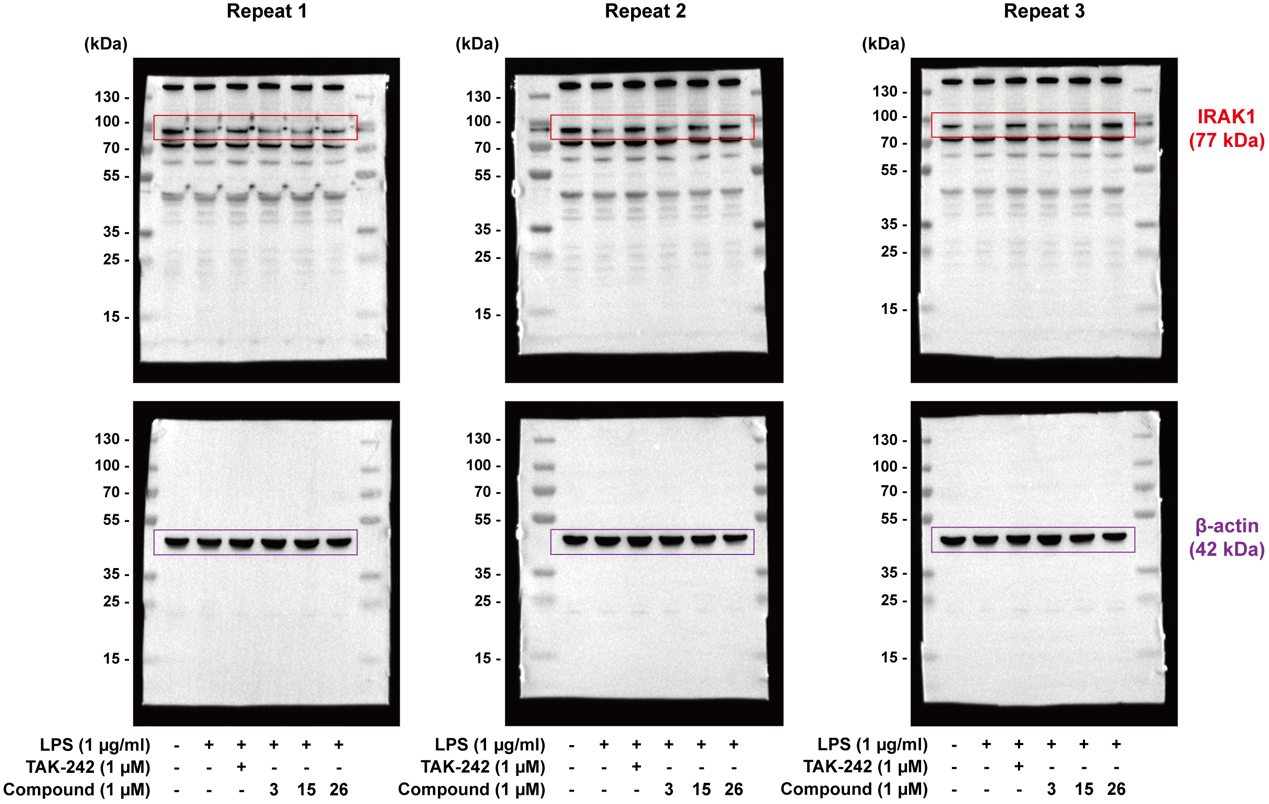
**

**Fig. S13** Original Western blots of IRAK1 in Fig. 5E, and β-actin was selected as loading control (*n* = 3).

**
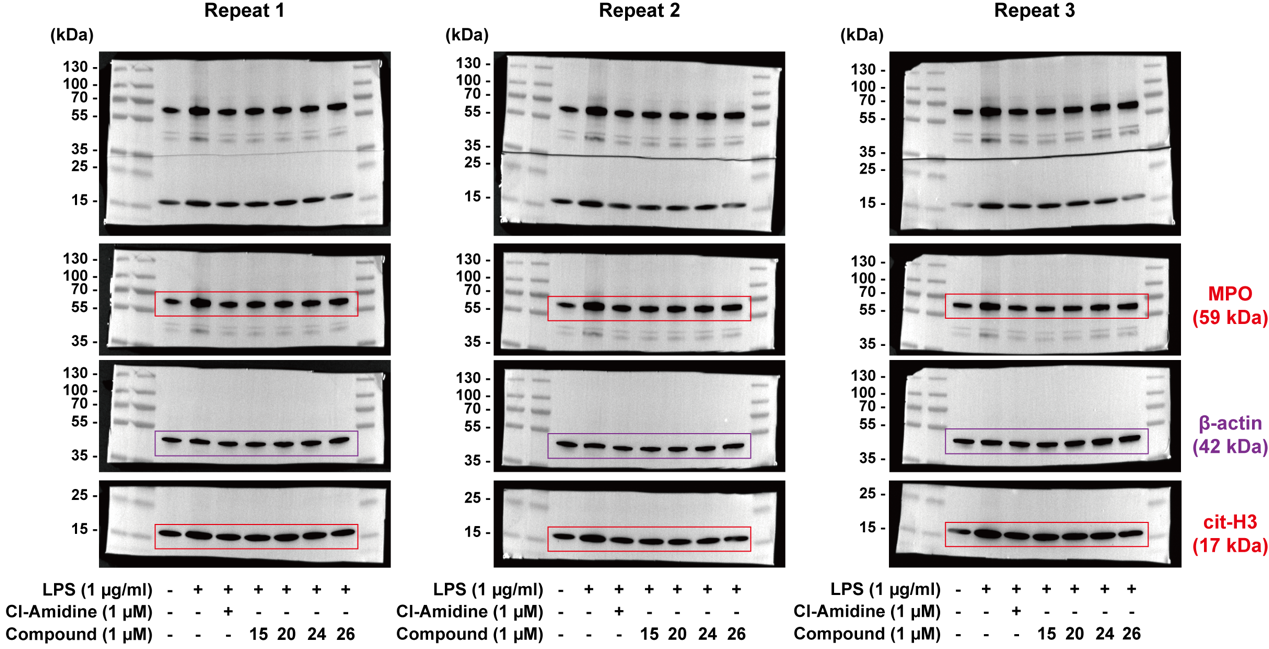
**

**Fig. S14** Original Western blots of MPO and cit-H3 in Fig. 6B, and β-actin was selected as loading control (*n* = 3).

**
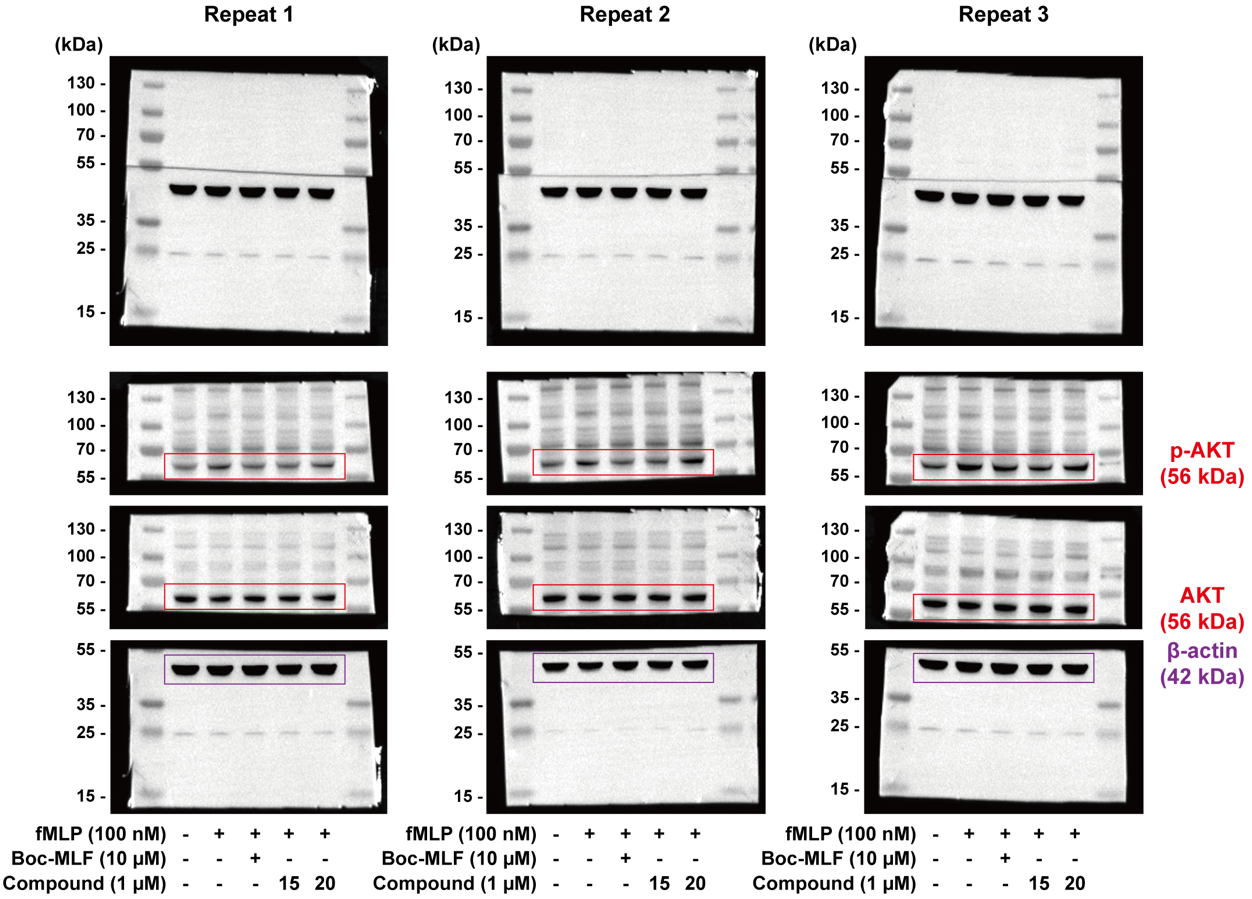
**

**Fig. S15** Original Western blots of AKT and p-AKT in Fig. 6D, and β-actin was selected as loading control (*n* = 3).

**
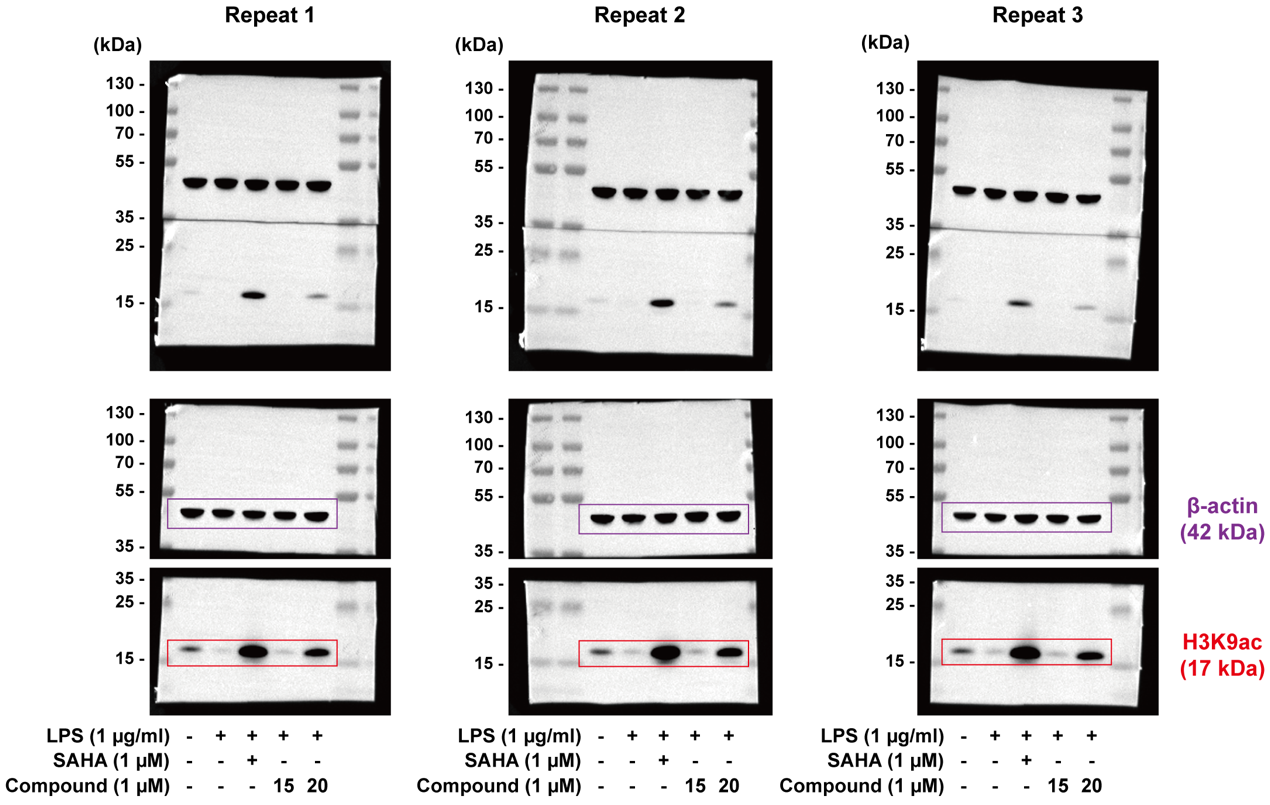
**

**Fig. S16** Original Western blots of H3K9ac in Fig. 6E, and β-actin was selected as loading control (*n* = 3).

**Methods：**

**Method S1 UPLC/Q-TOF-MS/MS analysis for FZJD extract and plasma sample**

A Waters Acquity UPLC System (Waters, Co., USA) equipped with a photodiode array detector and a Waters Q/TOF Premier Mass Spectrometer with an electrospray ionization system (Waters MS Technologies, Manchester, UK) were used for the sample analysis. The data acquisition was supported by using the Mass Lynx V4.1 software (Waters Co., USA).

An ACQUITY UPLC BEHC18 chromatographic column (2.1 mm × 100 mm, 1.7 μm; Waters Corporation, Milford, MA, USA) was used and eluted with a linear gradient of A (0.1 formic acid in water) and B (acetonitrile) at a flow rate of 0.3 ml/min and a temperature of 25°C. The gradient program was as follows: initial, 5% B; 3.0 min, 10% B; 4.0 min, 18% B; 10.0 min, 22% B; 13.0 min, 24% B; 18.0 min, 26% B; 20.0 min, 28% B; 23.0 min, 45% B; 26.0 min, 65% B; 29.0 min, 95% B; 31.0 min, 5% B; and 35.0 min, 5% B.

Mass spectrometry was performed using a Waters Q-TOF Premier with electrospray ionization (ESI) system (Waters MS Technologies, Manchester, UK) operating in both positive and negative modes. The electrospray capillary voltage was set to 3.0 kV for the positive mode and 2.5 kV for the negative mode. The sample cone voltage was set to 30 V, the nebulization gas was set to 600 L/h at 250°C, the cone gas set to 50 L/h, and the source temperature was 100°C. The Q-TOF Premier acquisition rate was 0.1 s with a 0.02 s inter-scan delay. The MS spectra were acquired from 100 to 1500 Da.

**Method S2 Quantitative proteomics and non-targeted metabolomics analysis**

For quantitative proteomics, proteins were extracted from liquid nitrogen-flash frozen samples using urea lysis buffer (8 M urea, 1 mM PMSF, 2 mM EDTA) with ice-cooled ultrasonication. After centrifugation (15000 g, 4°C, 10 min), supernatants were quantified via BCA assay. For digestion, proteins were reduced (10 mM DTT, 37°C, 45 min), alkylated (50 mM iodoacetamide, 25°C, 15 min), acetone-precipitated, and trypsin-digested (25 mM NH_4_HCO_3_, 37°C, overnight). Peptides were desalted using C18 cartridges.

LC-MS/MS analysis was performed on a timsTOF Pro2 system (Bruker) coupled to nanoElute UHPLC with Aurora C18 column (25 cm × 75 μm). Mobile phase B (0.1% FA in ACN) increased from 2% to 80% over 40 min. diaPASEF mode employed 48 isolation windows (400-1200 m/z) with mobility-dependent collision energy (27-45 eV). Data were processed through DIA-NN (v1.8.1) against UniProt mouse database (55,319 entries) with 1% FDR threshold.

As for metabolomics, samples were extracted with Acetonitrile : MeOH (1:4) containing internal standards. Dual LC-MS runs (positive/negative modes) used HSS T3 column (2.1 × 100 mm, 1.8 μm) with 0.4 ml/min flow and identical gradient: 5-99% B (0.1% FA-ACN) over 7.5 min. Sciex 5600+ Q-TOF operated in IDA mode (DP ±60 V, ISVF ±5000 V). Differential metabolites were identified by VIP > 1 and *p* < 0.05 (t-test/ANOVA). Pathway enrichment was performed via hypergeometric test using KEGG annotations.
